# Wild-Grown Romanian *Eupatorium cannabinum*: Advancing Phyto-Nanocarriers via Maltodextrin Micro-Spray Encapsulation—Metabolite Profiling, Antioxidant, Antimicrobial, and Cytotoxicity Insights

**DOI:** 10.3390/polym17040482

**Published:** 2025-02-12

**Authors:** Gabriela Vlase, Adina-Elena Segneanu, Ludovic Everard Bejenaru, Ionela Amalia Bradu, Crina Sicoe, Titus Vlase, George Dan Mogoşanu, Gabriela Buema, Dumitru-Daniel Herea, Maria Viorica Ciocîlteu, Cornelia Bejenaru

**Affiliations:** 1 Institute for Advanced Environmental Research, West University of Timişoara (ICAM–WUT), 4 Oituz Street, 300086 Timişoara, Timiş County, Romania; gabriela.vlase@e-uvt.ro (G.V.); adina.segneanu@e-uvt.ro (A.-E.S.); ionela.bradu@e-uvt.ro (I.A.B.); titus.vlase@e-uvt.ro (T.V.); 2 Research Center for Thermal Analyzes in Environmental Problems, West University of Timişoara, 16 Johann Heinrich Pestalozzi Street, 300115 Timişoara, Timiş County, Romania; 3 Department of Pharmacognosy & Phytotherapy, Faculty of Pharmacy, University of Medicine and Pharmacy of Craiova, 2 Petru Rareş Street, 200349 Craiova, Dolj County, Romania; george.mogosanu@umfcv.ro; 4 Faculty of Chemistry, Biology, Geography, West University of Timişoara, 16 Johann Heinrich Pestalozzi Street, 300115 Timişoara, Timiş County, Romania; crinandreea89@gmail.com; 5 National Institute of Research and Development for Technical Physics, 47 Dimitrie Mangeron Avenue, 700050 Iaşi, Iaşi County, Romania; gbuema@phys-iasi.ro (G.B.); dherea@phys-iasi.ro (D.-D.H.); 6 Department of Instrumental and Analytical Chemistry, Faculty of Pharmacy, University of Medicine and Pharmacy of Craiova, 2 Petru Rareş Street, 200349 Craiova, Dolj County, Romania; maria.ciocilteu@umfcv.ro; 7 Department of Pharmaceutical Botany, Faculty of Pharmacy, University of Medicine and Pharmacy of Craiova, 2 Petru Rareş Street, 200349 Craiova, Dolj County, Romania; cornelia.bejenaru@umfcv.ro

**Keywords:** *Eupatorium cannabinum*, gold nanoparticles, phytocomplex, secondary metabolites, micro-spray encapsulation, antioxidant potential, antimicrobial screening, in vitro cytotoxicity

## Abstract

In Romanian ethnopharmacology, *Eupatorium cannabinum* species is known for its remarkable biological activity. We present an advanced approach to encapsulation using maltodextrin matrices to enhance the stability and efficacy of phytoconstituents and nanoparticles. Two distinct carrier systems were developed: (i) a direct micro-spray encapsulation of *E. cannabinum* in maltodextrin to produce a maltodextrin-encapsulated carrier (MEC), and (ii) a two-step process involving the preparation of a new phytocarrier system based on gold nanoparticles (EC-AuNPs), followed by micro-spray encapsulation in maltodextrin to create the maltodextrin-encapsulated AuNPs system (MEC-AuNPs system). Comprehensive chemical profiling using GC–MS and ESI–QTOF–MS revealed 80 bioactive molecules, including terpenoids, alkaloids, flavonoids, and phytoecdysteroids. Morpho-structural (XRD, FTIR, Raman spectroscopy, SEM) and thermal analyses confirmed the successful integration of NPs within the matrices. EC-AuNPs and MEC-AuNPs exhibited superior antioxidant activity, significant antimicrobial efficacy against major bacterial pathogens (*S. aureus*, *B. subtilis*, *B. cereus*, *P. aeruginosa*, *S. typhi*, and *E. coli*), and enhanced cytotoxicity against MCF-7 and HT-29 cancer cell lines. This study highlights the potential of combining *E. cannabinum* with AuNPs and maltodextrin encapsulation to develop multifunctional therapeutic systems. The findings underscore the importance of phytoconstituent stabilization and nanotechnology in addressing global antimicrobial resistance and advancing innovative medical applications.

## 1. Introduction

*Eupatorium cannabinum* L., commonly known as hemp agrimony, is a perennial species within the *Asteraceae* family and represents the sole member of the *Eupatorieae* tribe indigenous to Romania [1,2,3]. This plant is typically encountered in moist habitats, particularly in riparian zones and along watercourses, and it is widely distributed across the country’s lowland and mountainous regions [1,2,3].

*E. cannabinum* can reach heights ranging from 50 to 175 cm, featuring a cylindrical, slightly hairy stem. The leaves are opposite, shortly petiolate, palmately divided into three to five leaflets, with lanceolate leaflets tapered at both ends and unevenly serrated margins. The flowers are arranged in compound racemose inflorescences (corymbs with capitula) positioned terminally. The capitula are 2–5 mm in diameter. The involucral bracts are unequal, imbricated, and finely pubescent or glabrous. All flowers are hermaphroditic, tubular, and pentamerous, displaying colors ranging from red and pink to white. The fruits are achenes approximately 3 mm long, five-angled, black, and equipped with a pappus of simple, uniseriate hairs. In Romania, flowering occurs from July to September [1,2,3,4,5].

The *Eupatorium* genus is characterized by a diverse array of phytochemicals, which include essential oil (EO), flavonoids, phenylpropanoids, pyrrolizidine alkaloids (PAs), tannins, terpenoids (including mono-, sesqui-, di-, and triterpenoids), and polysaccharides, with the total number of identified compounds surpassing 300 [6,7]. Among the most extensively studied species are *E. perfoliatum*, *E. chinense*, *E. arnottianum*, and *E. lindleyanum*, which have been documented to exhibit anti-inflammatory, antinociceptive, antibacterial, antifungal, antioxidant, cytostatic, and cytotoxic properties [6,7].

*E. cannabinum* holds a significant place in traditional phytotherapy across various cultures, where it is utilized for its laxative, diuretic, choleretic-cholagogue, and hypocholesterolemic effects. Additionally, it is incorporated into local rituals and employed in various conditions treatment such as hepatitis, hypertension, headaches, diabetes mellitus, influenza, colds, fever, and wound healing. In Europe, *E. cannabinum* is valued for its choleretic, diuretic, laxative, and hypocholesterolemic properties and for its effectiveness in addressing skin conditions like furuncles, eczema, and psoriasis [5,8,9,10].

While *E. cannabinum* has a history of traditional use, research on its chemical composition and pharmacological actions has been relatively limited. Efforts to identify the chemical compounds in *E. cannabinum* have primarily focused on detecting EO, flavonoids, phenolic acids, PAs, and polysaccharides [5,11,12,13,14,15,16,17].

Research efforts utilizing extracts, isolated compounds, and EO from the aerial parts of the plant have confirmed various pharmacological activities, including immunomodulatory, antioxidant, hepatoprotective, anti-inflammatory, antimicrobial, and cytostatic effects [7,10,14,16,17,18,19].

The studies conducted on *E. cannabinum*, specifically evaluating its antiproliferative activity using polar and non-polar extracts as well as pure compounds like sesquiterpene lactones (eupatoriopicrin), are significant in understanding its potential therapeutic benefits in oncological applications. In vitro tests on human tumor cell lines (HT29, BT-20, HepG2, Caco-2) and in vivo studies on laboratory animals (mouse models) have provided valuable insights into the efficacy of *E. cannabinum* [18,19,20,21,22,23]. These efforts contributed to a more comprehensive understanding of the potential role of *E. cannabinum* in cancer treatment.

The increasing prevalence of antimicrobial resistance (AMR) and the global burden of cancer highlight the urgent need for innovative and effective therapeutic solutions. Addressing these challenges requires ongoing research and development efforts to combat AMR and improve cancer treatment outcomes. Multidrug-resistant (MDR) bacteria, such as *Escherichia coli*, *Pseudomonas aeruginosa*, and *Staphylococcus aureus*, are becoming increasingly untreatable with conventional antibiotics. Projections estimate AMR will result in up to 10 million annual deaths by 2050, highlighting an urgent need for new antimicrobial agents [24]. Similarly, cancer, with 19.3 million new cases in 2020 alone, requires the development of more selective and efficient treatments to minimize side effects and improve patient outcomes [25].

Nanotechnology offers a transformative approach to addressing these challenges. Gold nanoparticles (AuNPs) are widely studied for their applications in medicine, with two primary synthesis methods dominating: chemically synthesized citrate-coated AuNPs and green-synthesized AuNPs. Notably, citrate-coated AuNPs are renowned for their tunable size and stability, rendering them suitable for various biomedical applications, such as cancer therapy, biosensing, and tissue engineering [26]. The citrate coating not only serves to stabilize the particles but also enables functionalization with bioactive compounds, enhancing targeted drug delivery and imaging capabilities [26,27,28,29,30,31,32]. These NPs exhibit broad-spectrum antimicrobial activity, disrupting bacterial membranes and interfering with microbial deoxyribonucleic acid (DNA). Moreover, they demonstrate antitumoral effects by generating reactive oxygen species (ROS) and inducing apoptosis in cancer cells [26,27,28,29,30,31,32].

To this end, developing engineered herbal carriers using NPs is a cutting-edge advancement in biomedical research [33]. Medicinal plants, rich in bioactive phytoconstituents with therapeutic potential, often face stability, bioavailability, and specificity challenges [33,34,35,36,37]. Additionally, the levels of secondary metabolites present in these plants are influenced by various factors, including abiotic and biotic conditions, growth stages, and parameters related to extraction techniques, such as temperature, solvent polarity, duration, and pH [38,39,40]. These factors collectively dictate the chemical profile and biological activity of the herb.

NP-based delivery systems enhance the biological properties of phytoconstituents and enable targeted therapeutic effects. Recent research emphasizes the importance of such tailored scaffolds in specific targeting and localization on biological surfaces, improving efficacy and minimizing off-target effects [39,40,41,42]. Furthermore, nanotechnology in herbal formulations amplifies phytoconstituent properties, aiding in combating MDR pathogens and targeting tumor cells [38,39,40,41,42]. This dual functionality showcases the potential of NP-based herbal formulations in addressing global health issues like AMR and cancer [38,39,40,41,42]. To further improve the applicability of NP-based systems, tailor-made carriers are being designed to enhance the stability, bioavailability, and targeted delivery of these therapeutic agents [38,39,40,41,42].

Maltodextrin, a safe and biodegradable polysaccharide, has gained attention as a NP encapsulation matrix due to its ability to protect bioactive agents, ensure sustained release, and improve solubility [43,44]. Advanced encapsulation techniques, such as spray drying, enable the precise formulation of stable carrier systems with augmented antimicrobial and cytotoxic effects [43,44,45].

This study investigates the development of a novel engineered herb–AuNP system through loading *E. cannabinum* with AuNPs, which is subsequently encapsulated in maltodextrin via spray drying. A comprehensive range of physical and chemical characterizations was conducted to assess the stability, morphology, and interaction between the phytoconstituents, AuNPs, and maltodextrin. Additionally, the biological efficacy of the system was evaluated through in vitro assessments of antioxidant activity, antimicrobial properties, and cytotoxicity potential. To the best of our knowledge, this study is the first to report on the low-metabolite profile of this wild-grown plant, thereby providing valuable insights into its bioactive potential.

## 2. Materials and Methods

### 2.1. Chemicals and Reagents

All reagents used in the study were of analytical grade. Methanol, ethanol, dichloromethane, chloroform, sodium carbonate, gallic acid, 2,2-diphenyl-1-picrylhydrazyl (DPPH), acetate buffer solution (pH 4–7), ferric reducing antioxidant power (FRAP) assay kit (MAK369-1KT), and dimethyl sulfoxide (DMSO) were sourced from Sigma Aldrich (Munich, Germany) and utilized without additional purification. The 3-(4,5-dimethylthiazol-2-yl)-2,5 diphenyl tetrazolium bromide (MTT) kit was obtained from AAT Bioquest (Pleasanton, CA, USA). Ultrapure water was employed in all experimental procedures. Maltodextrin (dextrose equivalents: 16.5–19.5) was obtained from Carbosynth (Berkshire, UK).

### 2.2. Cell Lines

The cell lines used in the study were obtained from the American Type Culture Collection (ATCC; Manassas, VA, USA). These cell lines were cultivated at 37 °C in Dulbecco’s Modified Eagle’s Medium (DMEM; Gibco, Life Technologies, Leicestershire, UK), supplemented with 10% fetal bovine serum (FBS) and 1% antibiotic–antimycotic solution (Sigma Aldrich).

### 2.3. Bacterial Strains

The bacterial strains used in the study were *Staphylococcus aureus* (ATCC 29213), *Bacillus subtilis* (ATCC 9372), *Bacillus cereus* (ATCC 14579), *Pseudomonas aeruginosa* (ATCC 27853), *Salmonella typhi* (ATCC 19430), and *Escherichia coli* (ATCC 25922), all of which were obtained from the ATCC (Manassas, VA, USA).

### 2.4. Plant Material

The *E. cannabinum* (EC) samples (whole plant—stems of 165 cm in height, leaves, flowers, and roots) were collected in July 2022 from the area of Baia de Aramă City, in the Southwest of Romania and taxonomically authenticated at the University of Medicine and Pharmacy of Craiova. Voucher specimens (EPT-CBN-2022-0712) were deposited at the Department of Pharmaceutical Botany, Faculty of Pharmacy, University of Medicine and Pharmacy of Craiova.

### 2.5. Preparation of AuNPs

AuNPs were prepared following a procedure outlined in a previous paper authored by the researchers [46,47].

### 2.6. Plant Sample Preparation for Chemical Screening

The freeze-dried plant samples (whole plant) were milled utilizing a planetary Pulverisette mill (Fritsch, Idar-Oberstein, Germany) at a speed of 780 rpm for 15 min, maintained at a temperature of 22 °C. The resulting milled material was then subjected to sieving through the American Society for Testing and Materials (ASTM) standard test sieve series [48] to isolate particles within the 0.20 to 0.25 mm range. Subsequently, the plant material underwent sonication extraction using an Elmasonic device (Elma Schmidbauer GmbH, Singen, Germany) for 45 min at a temperature of 38 °C and a frequency of 70 Hz, dissolved in 23 mL of methanol. All extracts were prepared in triplicate.

### 2.7. GC–MS Analysis

The gas chromatography (GC) analysis was conducted using the GCMS-QP2020 NX Shimadzu equipment with a ZB-5MS capillary column from Agilent Technologies (Santa Clara, CA, USA). The column specifications include a length of 30 m, an inner diameter of 0.25 mm, and a film thickness of 0.25 μm. Helium was used as the carrier gas at 1 mL/min flow rate.

The oven temperature program was initially at 45 °C, where it was held for 2.5 min, then increased at a rate of 3 °C per minute to a final temperature of 310 °C, which was maintained for five minutes. The injector temperature was set to 275 °C, and the interface temperature to 225 °C. Compound masses were measured using an ionization energy of 80 eV, with data acquisition starting after a one-minute solvent delay. The mass spectrometer source and MS Quad temperatures were maintained at 230 °C and 150 °C, respectively. Each analysis was performed in triplicate. Compound identification was performed by comparing their mass spectra with the National Institute of Standards and Technology (NIST) 2.0 software database (NIST, Gaithersburg, MD, USA), further supported by a review of the relevant literature. To refine the identification process, retention indices (RIs) were calculated using the Van den Dool and Kratz formula, with a C_7_–C_40_ *n*-alkane mixture acting as the internal standard in the analytical sample. In parallel, the Kováts RI was determined via logarithmic interpolation, following the established equation for isothermal chromatographic conditions [49,50].

This meticulous methodology was selected to ensure accurate and reliable identification of phytoconstituents, while the incorporation of RIs strengthens the robustness and reproducibility of the results, offering greater confidence in their comparability.

### 2.8. MS Analysis

Mass spectrometry (MS) experiments were conducted using an electrospray ionization (ESI)–quadrupole-time-of-flight (QTOF)–MS analysis system (Bruker Daltonics, Bremen, Germany). Spectra were acquired in positive ion mode across a mass range of 50–3000 *m*/*z*, with a scan speed of 2.0 scans per second, collision energies ranging from 20 to 80 eV, and a source block temperature maintained at 80 °C. Phytoconstituents were identified by referencing the NIST/National Bureau of Standards (NBS)-3 library (NIST, Gaithersburg, MD, USA) [51] and by cross-validating the results with a comprehensive review of the relevant scientific literature.

### 2.9. Spray Drying Process

The spray-drying process was carried out utilizing a Mini Spray Dryer B-290 (Pilotech, Shanghai, China). The parameters were set with a feed flow rate of 8 mL/min, and the inlet and outlet temperatures were maintained at 120 °C and 72 °C, respectively. An inlet air flow rate of 35 m^3^/h was employed, along with a compressor air pressure of 0.06 MPa and a nozzle diameter of 0.7 mm. Additionally, the system operated with 100% suction airflow in an environment exhibiting approximately 80% humidity [52,53].

### 2.10. Phytocarrier System Preparation (EC-AuNPs System)

The EC-AuNPs system was prepared by combining *E. cannabinum* (solid herb samples, prepared as previously described) with an AuNPs solution in a 1:5 mass ratio. The mixture was subjected to ultrasonic mixing for 40 min at 45 °C, followed by filtration using F185 mm filter paper. The filtrate was then dried in an oven at 50 °C for eight hours. Each experiment was performed in triplicate to ensure reproducibility.

### 2.11. Preparation of Maltodextrin–E. cannabinum Carrier (MEC Carrier)

To prepare the first carrier (MEC), 1.5 g of dried *E. cannabinum* sample and 1.5 g of maltodextrin were mixed, dissolved in 50 mL of ultrapure water, and thoroughly homogenized. The solution was incubated at 40 °C for 30 min with continuous stirring, followed by centrifugation for eight minutes and filtration using Whatman filter paper (0.45 μm). The filtered solution was then processed through the spray dryer, and the resulting dried powder was carefully collected and stored in an opaque, airtight container at 22 °C until further analysis. All experiments were conducted in triplicate.

### 2.12. Preparation of Maltodextrin–EC-AuNPs System (MEC-AuNPs System)

Following the same experimental procedure, the MEC-AuNPs system was prepared from the EC-AuNPs system and maltodextrin in a 1:1 mass ratio. All experiments were conducted in triplicate.

### 2.13. Characterization of Carriers

#### 2.13.1. FTIR Spectroscopy

Fourier-transform infrared (FTIR) data were collected using a Shimadzu AIM-9000 spectrometer equipped with attenuated total reflectance (ATR) devices (Shimadzu, Tokyo, Japan). The spectra were recorded over 20 scans with a resolution of 4 cm^−1^ across the 4000–400 cm^−1^ range. Wavelength assignments were made based on a comprehensive review of the relevant literature.

#### 2.13.2. XRD Analysis

X-ray diffraction (XRD) analysis was conducted using a Bruker AXS D8-Advance X-ray diffractometer (Bruker AXS GmbH, Karlsruhe, Germany) with CuKα radiation (λ = 0.1541 nm), equipped with a rotating sample stage, an Anton Paar TTK low-temperature cell (−180 °C to 450 °C), high vacuum, inert atmosphere, relative humidity control, and an Anton Paar TTK high-temperature cell (up to 1600 °C). The resulting XRD patterns were systematically compared to the International Centre for Diffraction Data (ICDD) Powder Diffraction Database (ICDD file 04-015-9120). Furthermore, the average crystallite size and phase content were calculated using the whole-pattern profile fitting (WPPF) methodology.

#### 2.13.3. SEM Analysis

Scanning electron microscopy (SEM) micrographs were obtained using an SEM–energy dispersive X-ray spectroscopy (EDS) system (JSM-IT200 InTouchScope™ Scanning Electron Microscope, Freising, Germany) equipped with a field emission gun (FEG).

#### 2.13.4. DLS Particle Size Distribution Analysis

Dynamic light scattering (DLS) analysis was performed using a Microtrac/Nanotrac 252 (Montgomeryville, PA, USA), with each sample carried out in triplicate at room temperature (RT; 22 °C) and a scattering angle of 172°.

### 2.14. Encapsulation Efficiency, Loading Capacity and Encapsulation Yield

The encapsulation efficiency (*EE%*) was calculated as a percentage of the total amount (g) of *E. cannabinum* sample and EC-AuNPs system from the total amount (*g*) used as raw material in the encapsulation process, according to Equation (1) [52,53,54].

(1) $$EE\;\left(\%\right)=\frac{amount\;of\;compound\;encapsulated\;(g)}{amount\;compound\;used\;as\;raw\;materials\;(g)}\times 100$$

The loading capacity (*EC%*) was calculated as the ratio of the weight of nanocapsules without the amount of compounds used as raw material (*g*) and with the amount of compounds used as raw material (*g*), according to Equation (2) [52,53].

(2) $$EC\;\left(\%\right)=\frac{weight\;of\;nanocapsules\;(g)}{weigh\;of\;compound\;used\;as\;raw\;material+weigt\;of\;maldextrin\;(g)}\times 100$$

The encapsulation yield (*EY%*) was determined as the ratio of the weight of material nanoencapsulated into chitosan and the raw materials weight, according to Equation (3) [54]:

(3) $$EY\;\left(\%\right)=\frac{weight\;of\;nanocapsules-amount\;of\;compound\;used\;as\;raw\;material\;(g)}{amount\;compound\;used\;as\;raw\;materials\;(g)}\times 100$$

The compound content and *EE%* of chitosan NPs were determined using an ultraviolet–visible (UV–Vis) Perkin-Elmer Lambda 35 (Perkin Elmer, Waltham, MA, USA). All absorbance measurements were taken in a 10 mm UV–Vis spectroscopy cell at RT, using a solvent (ethanol–chloroform 1:1, *v*/*v*) as a blank. The samples (15 mg) were subjected to sonication extraction (frequency of 50 kHz) in 20 mL of solvent (hydrochloric acid/ethanol/chloroform 3:2:2, *v*/*v*/*v*) for 40 min at RT (22 °C) and centrifuged. Subsequently, the supernatant concentration was determined by atomic UV–Vis spectroscopy. Each experiment was repeated three times [54].

### 2.15. Estimation of Total Phenolic Content and Antioxidant Activity

The total phenolic content (TPC) in *E. cannabinum*, the EC-AuNPs system, the MEC carrier, and the MEC-AuNPs system was determined using the Folin–Ciocalteu assay. The antioxidant activity of *E. cannabinum*, the EC-AuNPs system, the MEC carrier, and the MEC-AuNPs system was assessed through FRAP and DPPH assays. All antioxidant activity experiments were conducted in triplicate to ensure reproducibility.

#### 2.15.1. Sample Preparation Procedure

An aliquot of 0.25 g of *E. cannabinum* was added to 10 mL of 70% ethanol, and the mixture was stirred for eight hours at RT (22 °C). Afterward, the mixture was centrifuged at 5000 rpm for 10 min, and the resulting supernatant was collected for further evaluation of antioxidant potential. The same experimental procedure was applied to the EC-AuNPs system, MEC carrier, and MEC-AuNPs system.

#### 2.15.2. TPC Assay

The TPC of *E. cannabinum*, the EC-AuNPs system, the MEC carrier, and the MEC-AuNPs system samples, prepared as described above, was determined spectrophotometrically using a FLUOstar Optima UV–Vis spectrometer (BMG Labtech, Offenburg, Germany) following the Folin–Ciocalteu procedure adapted from our previous publications [54,55]. The results were expressed in gallic acid equivalents (mg GAE/g sample). Sample concentrations were calculated based on the linear Equation (4) derived from the standard curve, with a correlation coefficient (*R*^2^ = 0.9998):

*y* = 0.0023*x* + 0.0545 (4)

#### 2.15.3. FRAP Assay

The FRAP antioxidant activity of *E. cannabinum*, the EC-AuNPs system, the MEC carrier, and the MEC-AuNPs system samples was assessed spectrophotometrically using a FLUOstar Optima UV–Vis spectrometer (BMG Labtech) at 595 nm, employing a FRAP assay kit, following the procedure outlined in our earlier publication [47,55]. The results were expressed as mM Fe^2^⁺, calculated using the following Equation (5):

(5) $$\mathrm{FRAP}=\frac{C_{{Fe}^{2+}\;}\times F}{V}$$

where $C_{{Fe}^{2+}\;}$ is the amount (nM) of iron (*Fe*^2^⁺) ions generated from the calibration curve for each sample (nM), F is the dilution factor, and *V* is the volume of the sample (μL).

#### 2.15.4. DPPH Radical Scavenging Assay

The DPPH radical scavenging activity of *E. cannabinum*, the EC-AuNPs system, the MEC carrier, and the MEC-AuNPs system samples was evaluated according to the procedure described in our earlier publication [54,55]. Absorbance was recorded at 520 nm using a FLUOstar Optima UV–Vis spectrometer (BMG Labtech). The half-maximal inhibitory concentration (IC_50_) values (μg/mL) were determined from the inhibition percentage, *Inh (%)*, calculated using the calibration curve for each sample, as per Equation (6):

(6) $$\mathrm{Inh}\;(\%)=\frac{\left(A_{0\;\;}-A_{1}\right)}{A_{0}}\times 100$$

where *A*_0_ is the absorbance of the control and *A*_1_ is the absorbance of the sample.

### 2.16. Antimicrobial Activity

The antimicrobial activity of *E. cannabinum*, the EC-AuNPs system, the MEC carrier, and the MEC-AuNPs system samples was evaluated using agar well diffusion assay, minimum inhibitory concentrations (MICs), and minimum bactericidal concentrations (MBCs). MICs and MBCs were determined by the microbroth dilution method (Müller–Hinton medium). The MIC was defined as the lowest concentration of the compound that inhibits bacterial growth, and the MBC was the lowest concentration at which no visible bacterial growth occurred after a 14 h incubation.

Microbial growth inhibition was evaluated by measuring the optical density at 600 nm using a T90+ UV–Vis spectrophotometer (PG Instruments, Lutterworth, UK) [56]. The nutrient agar and nutrient broth were prepared following the manufacturer’s instructions and autoclaved at 125 °C for 25 min. The final concentration of microorganisms was adjusted to the 0.5 McFarland Standard (1.5 × 10^8^ CFU/mL; CFU: Colony-forming units) [57]. The samples were tested in triplicate.

Dilutions of five concentrations (100, 125, 150, 175, and 200 μg/mL) were prepared using 25% DMSO [56]. The agar well diffusion method was used to evaluate the antimicrobial potential, following the procedure described in our previous publication [55].

Each test was performed in triplicate [58,59,60].

### 2.17. Cell Culture Procedure

#### 2.17.1. Cell Culture and Treatment

This study employed MCF-7 (breast), and HT-29 (colon) cancer cell lines obtained from ATCC (Manassas, VA, USA). The cells were cultured at an incubation temperature of 37 °C with 5% CO_2_ and maintained at 100% humidity in DMEM supplemented with FBS and a 1% antibiotic–antimycotic solution. Following seeding at a density of 4 × 10^3^ cells per well in 96-well plates, the cells were incubated for 24 h to achieve approximately 90% confluency. The medium was then replaced with a fresh culture medium. Afterward, the medium was replaced with fresh culture medium containing various concentrations (75, 100, 125, 150, 175, and 200 μg/mL) of *E. cannabinum*, the EC-AuNPs system, the MEC carrier, and the MEC-AuNPs system. The cells were then incubated for an additional 24 h. A control group comprising fresh standard medium, as well as positive and negative controls, was included, with each treatment condition performed in triplicate. After the 24 h incubation period at 37 °C under 5% CO_2_, the viability of the cells was assessed.

#### 2.17.2. MTT Assay

Cell viability was assessed by aspirating the test materials from each well of the initial plate. Then, 25 μL of MTT reagent was added to each well, followed by two hours of incubation at 37 °C in a CO_2_ incubator. Afterward, the formazan crystals formed were solubilized with DMSO, and the absorbance was measured at 540 nm using a Multi-Mode Microplate Reader Synergy HTX spectrophotometer (Agilent Technologies, Santa Clara, CA, USA).

Cell viability was calculated using the following Equation (7):

(7) $$\mathrm{Inh}\;(\%)=\frac{\left(A_{0\;\;}-A_{1}\right)}{A_{0}}\times 100$$

where $C_{V\;}$(%) is the cell viability and ${\;O}_{Ds},\;O_{Db,\;}{\;O}_{Ds\;}$ denote the optical density of the wells containing cells with the test sample (OD_sample), only cells (OD_control), and cell culture media without cells (OD_blank).

The positive control consisted of untreated cells, MTT solution, and DMSO, while the negative control included only dead cells, MTT solution, and DMSO. The IC_50_ values were determined based on the concentrations (75, 100, 125, 150, 175, and 200 μg/mL) at which the test samples (*E. cannabinum*, the EC-AuNPs system, the MEC carrier, and the MEC-AuNPs system) exhibited 50% cell viability for MCF-7 and HT-29 cell lines. The data were plotted, and IC_50_ values were derived accordingly.

### 2.18. Statistical Analysis

All experiments, encompassing sample measurements, calibration curves, and concentration determinations, were conducted in triplicate. The data are presented as mean ± standard deviation (SD). Statistical analyses were performed using Student’s *t*-test within Microsoft Office Excel 2019 (Microsoft Corporation, Redmond, WA, USA). For multiple comparisons, Dunnett’s *post hoc* test was applied after a one-way analysis of variance (ANOVA). A *p*-value of less than 0.05 was deemed to indicate statistical significance.

## 3. Results

### 3.1. GC–MS Analysis of E. cannabinum

The compounds within the *E. cannabinum* sample were systematically separated and analyzed utilizing GC–MS. The findings of this analysis are presented in Figure S1 and are further detailed in Table 1.

The GC–MS analysis identified 24 compounds, accounting for 84.05% of the total peak area in *E. cannabinum* sample (Figure S1).

### 3.2. MS Analysis

The mass spectrum depicted in Figure S2 reveals the presence of diverse biomolecules categorized into alkaloids, terpenes, fatty acids, flavonoids, phenolic acids, hydrocarbons, organic acids, esters, sterols, coumarins, phenylpropanoids, fatty alcohols, and other miscellaneous constituents, aligning with previously reported findings in the literature (Table 2) [5,10,16,17,18,72,73,74,75,76,77].

### 3.3. Chemical Screening

A total of 80 molecules identified through MS were appointed to various categories: terpenoids (25.00%), flavonoids (16.25%), alkaloids (11.25%), fatty acids (6.25%), phytosterols (5.00%), phenolic acids (2.50%), esters (3.75%), hydrocarbons (7.50%), fatty alcohols (3.75%), aldehydes (6.25%), phenylpropanoids (1.25%), phytoecdysteroids (1.25%), coumarins (1.25%), and miscellaneous.

**Figure 1:**
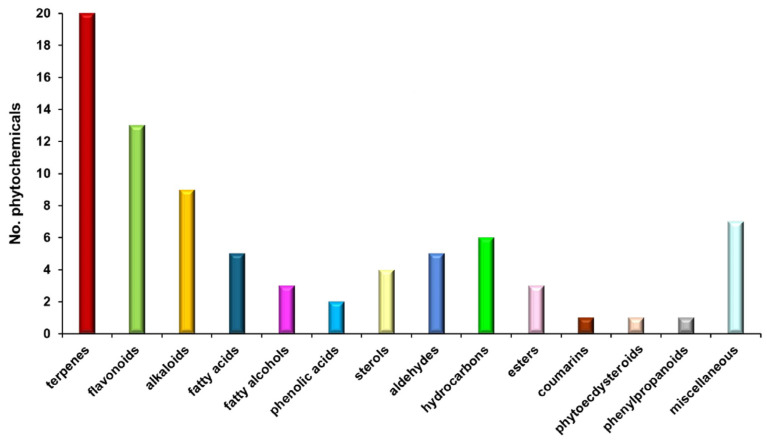
Biomolecules classification bar chart of *E. cannabinum* sample.

Figure 1 shows the classification bar chart of biomolecules from *E. cannabinum* according to the results of MS analysis (Table 2).

### 3.4. Key Aroma-Active Compounds Forming Different Flavor Characteristics

**Figure 2:**
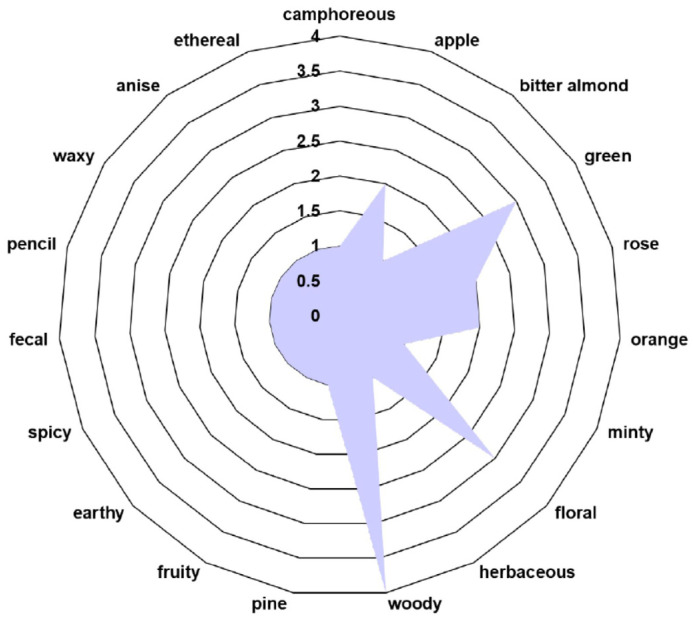
The volatile organic compound (VOC) odor profile of *E. cannabinum*.

The volatile organic compound (VOC) odor profile of phytochemicals identified in the *E. cannabinum* sample is shown in Table 3 and Figure 2.

### 3.5. Phytocarrier System

#### 3.5.1. FTIR Analysis

**Figure 3:**
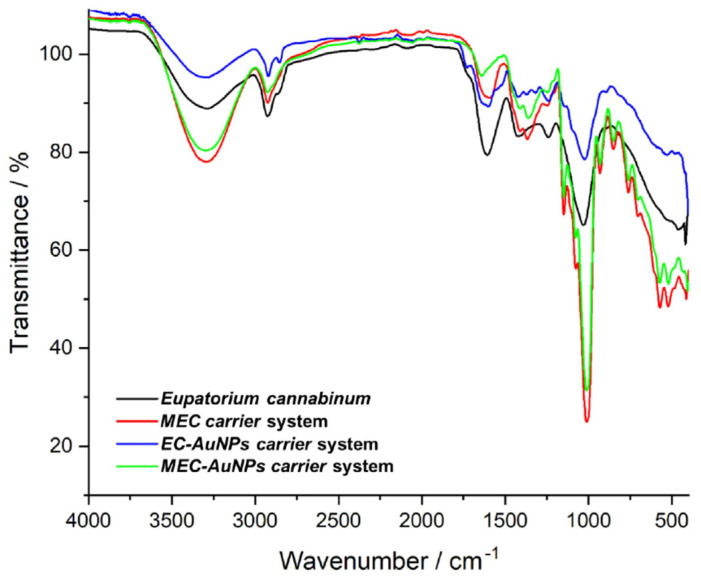
FTIR spectra of *E. cannabinum* sample, EC-AuNPs system, MEC carrier, and MEC-AuNPs system. EC-AuNPs, *E. cannabinum*–gold nanoparticles system; FTIR, Fourier-transform infrared; MEC, maltodextrin–*E. cannabinum*.

FTIR spectroscopy was applied to investigate the intricate chemical interplay between AuNPs and the diverse phytochemicals from *E. cannabinum* and to confirm the formation of the phytocarrier system (EC-AuNPs system). The FTIR analysis identified a spectrum of biomolecular classes, including terpenoids, alkaloids, flavonoids, fatty acids, coumarins, phenolic acids, phytosterols, aldehydes, esters, fatty alcohols, phytoecdysteroids, and phenylpropanoids. This compositional diversity highlights the multifaceted chemical landscape of the herb sample, presenting a unique array of molecular interactions with AuNPs (Figure 3; Table 4).

The FTIR spectra of the EC-AuNPs system display the vibrational peaks of phytoconstituents from *E. cannabinum* sample (Figure 3; Table 4), specifically at 3430 cm^−1^ (O–H stretching), 2922 cm^−1^ (–CH_2_ asymmetric vibration), 2854 cm^−1^ (CH– asymmetric and symmetric stretching), 1711 cm^−1^ (C=O stretching vibration), 1609 cm^−1^ (C=C of coumarin), 1637 cm^−1^ (C=O of flavonoids), 1603 cm^−1^ (C=C and N–H stretching vibrations of alkaloids), 1242 cm^−1^ (C–N of amine), 1034 cm^−1^ (NH stretching of amines), and 873 cm^−1^ (C–H bending vibration of aromatic rings), as well as the adsorption bands of characteristic to AuNPs coated with trisodium citrate: 2914 cm^−1^ (OH stretching vibration), 2846 cm^−1^ (corresponding to CH– asymmetric and symmetric stretching vibrations), 1595 cm^−1^ (COO– stretching vibration), and 1391 cm^−1^ (assigned to C–H bending), thus confirming the successful preparation of the EC-AuNPs carrier system [46,47,85,86].

Notably, the increased intensity of absorption bands and the shifts toward higher wavenumbers in the O–H, N–H, C–O, and C–H regions suggest active involvement of these functional groups in the EC-AuNPs carrier system preparation [46,86,87].

In the FTIR spectra of the MEC carrier, distinctive peaks characteristic of maltodextrin are evident, including the peak at 1024 cm^−1^ and 1152 cm^−1^ associated with C–O–C stretching of the glycosidic bond, the peak at 926 cm^−1^ corresponding to the α-1,4 glycosidic bond, and 848 cm^−1^ (C–H bending) [88,89]. Additionally, peaks representing various plant biomolecules are also visible.

Similarly, the MEC-AuNPs display the maltodextrin absorption bands and plant phytoconstituents. However, changes in the intensity of the absorption bands in the –OH and N–H regions, along with the emergence of two new peaks at 1429 cm^−1^ and 899 cm^−1^ in both MEC and MEC-AuNPs, provide strong evidence for the successful encapsulation of EC and the corresponding EC-AuNPs system within the biopolymeric matrix [90].

#### 3.5.2. XRD Analysis

**Figure 4:**
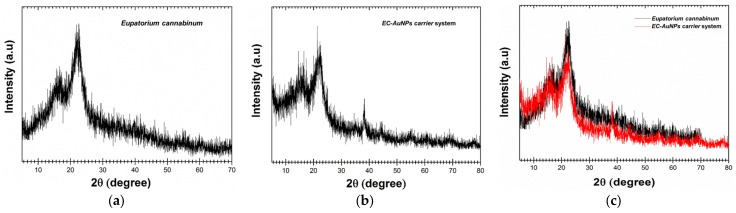
Powder XRD patterns of *E. cannabinum* sample (**a**), EC-AuNPs system (**b**), and overlapping XRD patterns of *E. cannabinum* and EC-AuNPs system (**c**). XRD, X-ray diffraction.

The comparative XRD analysis of *E. cannabinum* and the EC-AuNPs system is illustrated in Figure 4a–c. In the *E. cannabinum* diffraction pattern (Figure 4a,c), the peak observed at approximately 16° 2θ, along with the prominent peak around 22°, suggests the presence of an amorphous or semicrystalline phase alongside the crystalline content of cellulose within the fiber [46,47,54,55,91].

Regarding the specific categories of phytoconstituents, the distinct peaks observed in the XRD analysis correspond well with the findings documented in the literature [46,47,54,55]. Terpenoids, for instance, are typically characterized by unique diffraction peaks within the 10–20° 2θ range, which reflect their molecular structure and crystallinity. Alkaloids, on the other hand, are identified by prominent peaks at approximately 14.5° and 18.5° 2θ, indicating the presence of specific nitrogen-containing compounds. Flavonoids are primarily detected in the 20–30° 2θ range, where their crystalline forms display characteristic reflections.

Furthermore, the presence of weaker but distinct peaks at 38.2°, 44.1°, 64.3°, and 78.3° corresponds to the (111), (200), (220), and (311) facets of the face-centered cubic (FCC) crystal lattice of AuNPs. These peaks align with the standard diffraction pattern of pure gold, as documented in Joint Committee on Powder Diffraction Standards (JCPDS) Card No. 04-0784, thereby confirming the successful formation of the EC-AuNPs system.

#### 3.5.3. SEM–EDX Analysis

**Figure 5:**
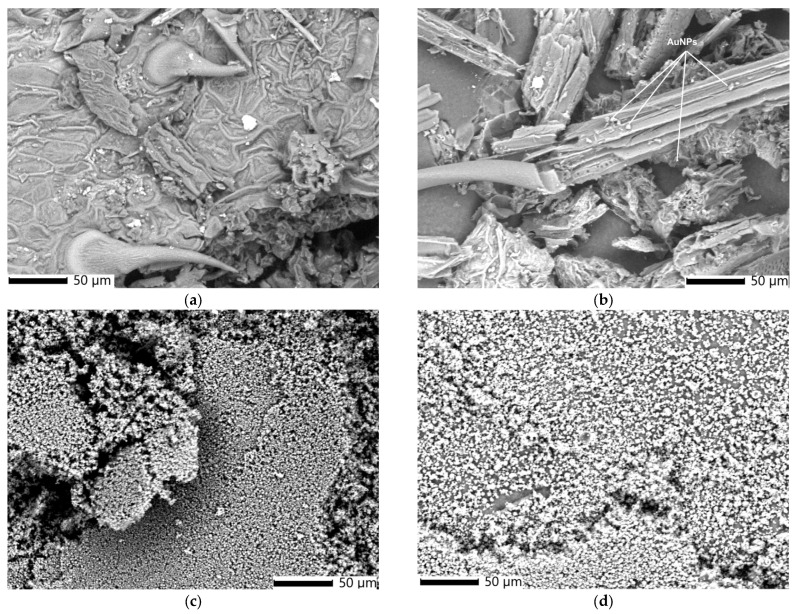
SEM image of *E. cannabinum* (**a**), EC-AuNPs system (**b**), MEC carrier system (**c**), and MEC-AuNPs carrier system (**d**). SEM, scanning electron microscopy.

Figure 5a–d presents scanning electron microscopy (SEM) micrographs illustrating the morphology of *E. cannabinum* and the EC-AuNPs system, both before and following encapsulation within the biopolymeric matrix.

The SEM image of *E. cannabinum* (Figure 5a) reveals a complex, textured surface structure characterized by prominent pores and unevenly distributed trichomes. This heterogeneous arrangement contributes to the plant’s microtopography, potentially enhancing the surface area for NP binding and stabilization.

Unlike the morphology of *E. cannabinum*, the EC-AuNPs system (Figure 5b) exhibits a fibrous structure with numerous spherical AuNPs (~18 nm) anchored to the fibers and loaded within the plant’s pores. These morpho-structural alterations result from the preparation process of the EC-AuNPs system.

After encapsulation, the SEM image of the MEC carrier (Figure 5c) indicates the clusters of spherical particles (~20 nm), suggesting effective encapsulation within the biopolymeric matrix.

The MEC-AuNPs micrograph (Figure 5d) reveals a dense distribution of uniformly sized, spherical particles (~30 nm) across the surface.

A notable observation after encapsulation in both the MEC carrier and MEC-AuNPs carrier systems is the significant reduction in particle size, accompanied by a more uniform particle distribution. Micrographs reveal substantial morphological transformations, with the biopolymeric matrix effectively encapsulating the particles from both the plant and EC-AuNPs systems. This encapsulation process results in a smoother, more homogenous texture within the matrix. The uniformity of these particles and their close-packed arrangement suggest highly effective encapsulation, which could enhance the structural integrity of the carrier systems and potentially improve their controlled release properties. These findings highlight the efficiency of the encapsulation process and its pivotal role in altering the structural and morphological characteristics of the biopolymeric matrix.

**Figure 6:**
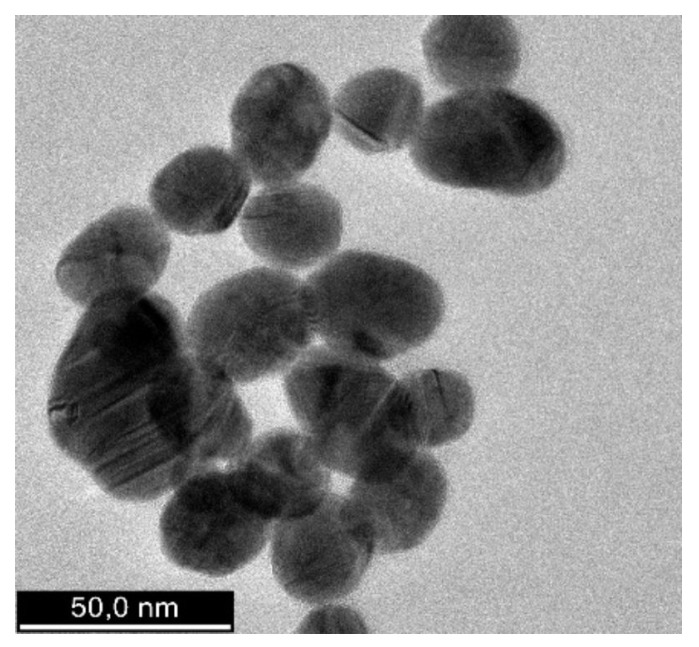
HR-TEM image of AuNPs. HR-TEM, high-resolution transmission electron microscopy.

The shape, morphology, and size distribution of the synthesized AuNPs were analyzed using transmission electron microscopy (TEM). The TEM image (Figure 6) reveals that the AuNPs are predominantly spherical, with average diameters ranging from 16 to 22 nm. This uniform shape and size distribution could enhance their stability and surface reactivity.

**Figure 7:**
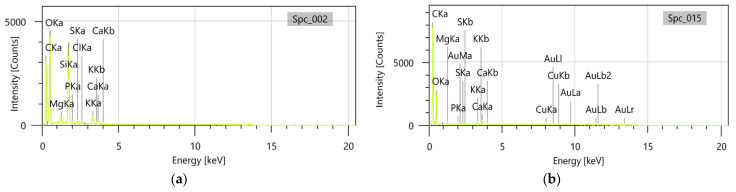
EDX analysis of the *E. cannabinum* (**a**) and EC-AuNPs system (**b**). EDX, energy dispersive X-ray.

Furthermore, the accompanying energy dispersive X-ray (EDX) analysis performed on *E. cannabinum* and the EC-AuNPs carrier system confirmed that, in the EC-AuNPs carrier system, peaks corresponding to both *E. cannabinum* (Figure 7a) and AuNPs (Figure 7b) were clearly observed, thereby verifying the successful preparation of the EC-AuNPs carrier system.

#### 3.5.4. DLS Analysis

**Figure 8:**
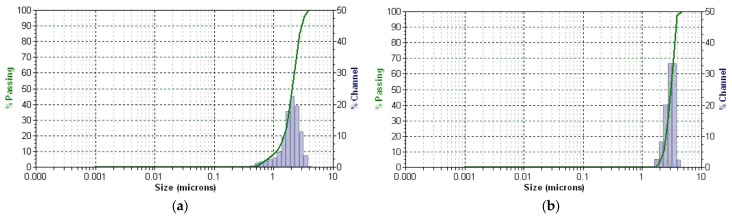
DLS patterns of *E. cannabinum* (**a**) and EC-AuNPs system (**b**). DLS, dynamic light scattering.

The plant particles and new EC-AuNPs system’s stability and dynamic behavior were analyzed using DLS. Figure 8a,b displays the results, illustrating particle size distribution (PSD) and temporal stability.

DLS profile of the *E. cannabinum* sample and the EC-AuNPs system reveals a single, distinct peak for each, indicating a monodisperse particle population in both samples. The mean diameter is 2.057 μm, with a polydispersity index (PDI) of 0.405 for the *E. cannabinum* plant particles, suggesting moderate size variability. In contrast, the EC-AuNPs system displays a slightly larger mean diameter of 3.050 μm and a lower PDI of 0.124, which reflects a more uniform PSD.

Moreover, the narrow peak observed in the EC-AuNPs system highlights its high stability, as this narrow distribution indicates minimal size variation within the particle population. This consistency suggests that the EC-AuNPs maintain a stable and homogeneous structure over time. These findings confirm that both systems possess stable, single populations of particles, with the EC-AuNPs system demonstrating enhanced uniformity and stability.

#### 3.5.5. PSD Analysis by Laser Diffraction

**Figure 9:**
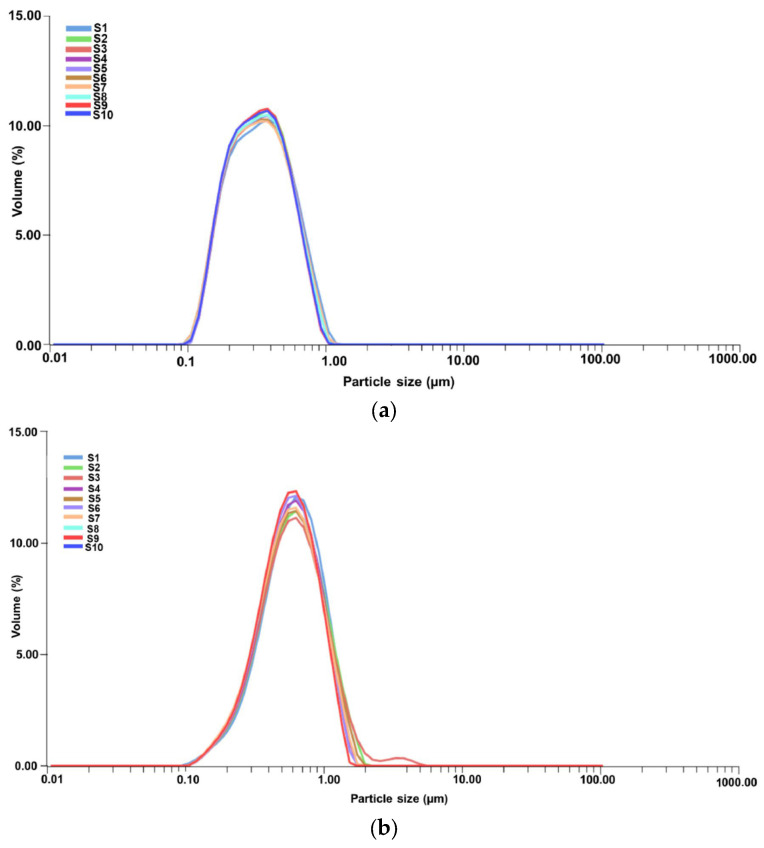
PSD curves for 10 consecutive measurements (taken over a 2 min period) for MEC carrier (**a**) and MEC-AuNPs system (**b**). PSD, particles size distribution.

The PSD of the encapsulated samples, specifically the MEC carrier and MEC-AuNPs, was assessed using laser diffraction analysis. The resulting data, presented in Figure 9a,b, provide insights into PSD and encapsulation consistency across the samples.

According to Figure 9a, each curve (S1 to S10) shows a consistent peak within a narrow range, indicating stable PSD over the duration of the measurements. This uniformity suggests that the MEC carrier particles maintain a stable dispersion without significant aggregation or size variation across the repeated scans.

Table 5 presents PSD metrics for the MEC and MEC-AuNPs samples, highlighting the effects of encapsulation. In this analysis, D[3,2] represents the surface-weighted mean diameter, while D[4,3] denotes the volume-weighted mean diameter. The biopolymeric capsules range from 0.380 μm for the MEC carrier to 0.649 μm for the MEC-AuNPs system in terms of D[4,3]. Similarly, the surface-weighted mean diameter (D[3,2]) is 0.298 μm for the MEC carrier and 0.521 μm for the MEC-AuNPs system. These results suggest that encapsulation decreases the mean diameter compared to the original *E. cannabinum* particles, as observed in the DLS analysis. Specifically, an 18.5% reduction in mean diameter for the MEC carrier and an even more substantial 21.3% decrease were observed in the EC-AuNPs system. This size reduction may indicate structural compaction or enhanced stabilization within the encapsulating matrix.

Conversely, the d_10_, d_50_, and d_90_ values correspond to particle diameters at the 10th, 50th, and 90th percentiles of the cumulative distribution, respectively [89,90]. For the MEC carrier, these values are 0.121 μm (d_10_), 0.312 μm (d_50_), and 0.714 μm (d_90_), indicating a relatively narrow size distribution [89,90]. In contrast, the MEC-AuNPs system exhibits d_10_, d_50_, and d_90_ values of 0.186 μm, 0.689 μm, and 1.123 μm, respectively, suggesting a broader particle size range in the encapsulated system [89,90]. This wider distribution implies that the encapsulation process introduces slight variability in particle size, likely due to the formation of a more complex composite structure around the EC-AuNPs within the maltodextrin matrix.

#### 3.5.6. Encapsulation Efficiency, Loading Capacity and Encapsulation Yield

EE%, EC%, and EY% are critical parameters for determining the quality of nanocapsules, their application potential, and economic feasibility [54,92].

The encapsulation data for the newly prepared phyto-nanocarriers are summarized in Table 6, highlighting the performance of the MEC carrier and MEC-AuNPs carrier systems.

The EY% values observed in this study align closely with values reported in the literature, further validating the efficiency of the encapsulation process [23,43,52]. For the MEC carrier, the EY% of 60.55% falls within the typical range for maltodextrin-based encapsulation systems, as noted in studies utilizing maltodextrin for encapsulating phytoconstituents and other bioactive compounds [23,43,52]. Similarly, the MEC-AuNPs system shows a slightly enhanced EY% of 61.27%, which can be attributed to the synergistic interplay between the unique properties of AuNPs, phytoconstituents, and the biopolymeric matrix interactions [93].

In the MEC carrier, these interactions are driven by a combination of hydrogen bonding, van der Waals forces, electrostatic interactions, and hydrophobic effects. Hydrogen bonding is established through the abundant –OH groups in maltodextrin, forming strong associations with the hydroxyl groups of phytoconstituents such as flavonoids, phenolic acids, and phenylpropanoids. Meanwhile, hydrophobic interactions occur between maltodextrin and the less polar biomolecules of *E. cannabinum*, including fatty acids, fatty alcohols, and phytosterols. Additionally, van der Waals forces contribute to the stability of the system by promoting interactions between the non-polar regions of phytochemicals and maltodextrin’s non-polar segments, ensuring effective integration of bioactive compounds into the biopolymeric matrix.

Conversely, the enhanced encapsulation parameters of the MEC-AuNPs system arise from the unique properties of AuNPs. Their high surface energy facilitates weak yet cumulative van der Waals interactions with maltodextrin, physically stabilizing the NPs. Furthermore, hydrogen bonding between maltodextrin’s hydroxyl groups and the ligands or surface coatings of AuNPs (such as phytoconstituents or citrate groups) contributes to their stability. Electrostatic interactions between maltodextrin’s polar regions and the charged surface of AuNPs promote uniform distribution and immobilization of the NPs, thereby enhancing structural integrity and encapsulation effectiveness. Additionally, they prevent the aggregation of AuNPs by creating a protective encapsulation barrier, which ensures even dispersion of NPs throughout the system. This encapsulation preserves the nanoscale properties of AuNPs, maintaining their optical, catalytic, and biological functionalities that would otherwise degrade upon aggregation. The resulting uniform distribution and stabilization not only protect the functional characteristics of AuNPs but also optimize their performance and reliability across a range of applications, making the MEC-AuNPs system an efficient and versatile platform.

### 3.6. Thermal Behavior Study

**Figure 10:**
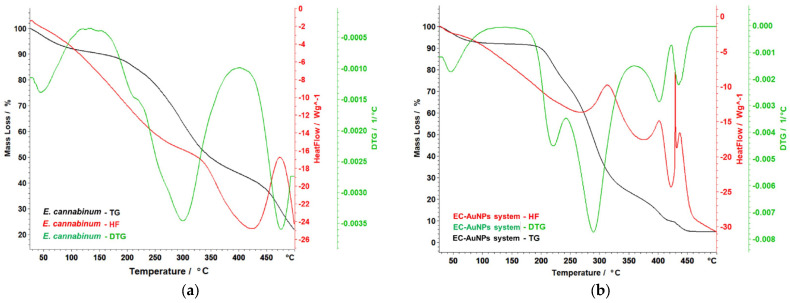

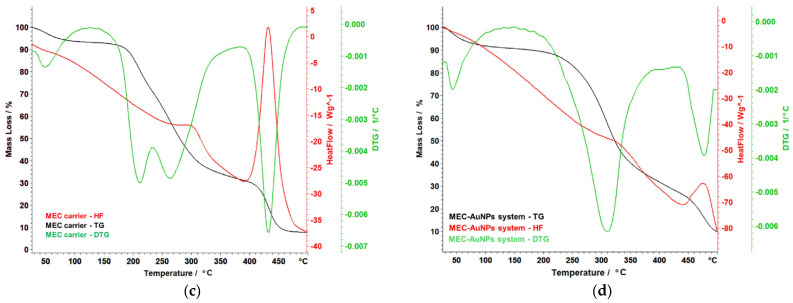
Themoanalytical curves of *E. cannabinum* sample (**a**), EC-AuNPs system (**b**), MEC carrier (**c**), and MEC-AuNPs system (**d**). DTG, differential thermogravimetry; HF, heat flow; TG, thermogravimetry.

The thermal behavior of *E. cannabinum* samples and the EC-AuNPs system was analyzed to evaluate their stability following encapsulation. The results of this analysis are illustrated in Figure 10a–d.

The thermal stability analysis of the samples (*E. cannabinum*, the EC-AuNPs system, the MEC carrier, and the MEC-AuNPs system) provides valuable insights into their characteristics and stability after encapsulation. Specifically, the findings for *E. cannabinum* reveal intricate thermal behavior consistent with its complex biochemical composition. As shown in Figure 10a, *E. cannabinum* undergoes a total mass loss of 75.18% during thermal decomposition. The initial mass loss of 6.43% occurs between 40 °C and 96 °C, attributed to moisture evaporation. This is followed by the degradation of organic materials, beginning with a primary degradation phase between 181 °C and 230 °C, where a mass loss of 6.22% is observed. This phase coincides with a peak in the differential thermogravimetry (DTG) curve at 208 °C, indicating a significant thermal reaction. Subsequently, a considerable mass loss of 34.63% occurs during a more pronounced degradation phase between 232 °C and 368 °C. This phase is marked by a peak on the DTG curve at 302 °C, associated with an exothermic reaction that suggests energy release during the breakdown of complex organic molecules. This decomposition likely corresponds to the thermal degradation of compounds such as hemicellulose, cellulose, polyphenols, polysaccharides, and tannins. At higher temperatures, around 433 °C, the degradation of more thermally stable compounds, such as lignin, becomes evident. This phase, characterized by another DTG curve peak at 474 °C, accounts for an additional mass loss of 17.66%. These findings underscore the complexity of *E. cannabinum*’s thermal degradation, highlighting the diverse organic constituents in its structure and their variable responses to thermal stress. In conclusion, the thermal stability analysis reveals the intricate thermal profile of *E. cannabinum,* demonstrating its susceptibility to decomposition across distinct temperature ranges.

The EC-AuNPs system demonstrates complex thermal behavior, as depicted in Figure 10b. An initial mass loss of 6.92% is observed up to 100 °C, which can be primarily attributed to the evaporation of water. The degradation of the organic phase commences between 181 °C and 244 °C, followed by a more intricate process that takes place between 245 °C and 354 °C. During this latter phase, a significant mass loss of 49.94% is recorded, with a notable peak on the DTG curve at 290 °C, accompanied by a pronounced exothermic effect peaking at 317 °C. Subsequent decomposition is observed between 390 °C and 427 °C, associated with the breakdown of more stable compounds or organic residues and marked by a DTG peak at 408 °C. The final decomposition phase, within the studied temperature range, extends up to 465 °C, resulting in an additional mass loss of 4.73%. Throughout these thermal events, slight shifts and variations in DTG peaks are evident, suggesting that AuNP’s presence may stabilize certain organic compounds, delaying or moderating their degradation.

Figure 10c illustrates the thermal behavior of the MEC carrier, highlighting distinct decomposition phases. Below 105 °C, moisture loss and the evaporation of volatile substances are observed. Between 173 °C and 332 °C, a complex process unfolds in two overlapping phases. The first phase involves a mass loss of 20.55%, with a DTG peak at 210 °C. The second phase, occurring between 235 °C and 332 °C, resulted in a mass loss of 34.65%, with a DTG peak at 267 °C. This stage corresponds to the decomposition of more volatile organic compounds and the initial degradation of maltodextrin. It is accompanied by an exothermic effect between 280 °C and 310° C. A subsequent decomposition phase occurs between 395 °C and 476 °C, with a mass loss of 22.85% and a DTG peak at 432 °C. This phase reflects the significant thermal degradation of the remaining organic structure, marked by a strong exothermic peak. The maltodextrin matrix serves as a protective barrier for the bioactive compounds but is ultimately degraded alongside the plant material during this stage.

The thermal behavior of the MEC-AuNPs (Figure 10d) system indicates a complex, multi-stage decomposition process characterized by significant mass loss, totaling 86.80%. The thermal analysis can be broken down into distinct temperature ranges with associated mass changes:

(i) Water loss (35–100 °C): This initial stage involves the loss of water, accounting for 7.45% of the total mass loss. This may suggest the presence of moisture in the system, likely associated with organic compounds or surface-bound water on the NPs;

(ii) Complex decomposition stage (205–390 °C): The most significant portion of the mass loss (55.38%) occurs within this temperature range. This stage likely corresponds to the breakdown of various organic compounds from this sample. The observation of a maximum on the DTG curve at 311 °C indicates a peak rate of decomposition, which is further supported by a strong exothermic effect observed between 320–340 °C. This exothermic behavior suggests vigorous chemical reactions occurring during decomposition, potentially leading to the formation of new products or the release of gases;

(iii) Late-stage decomposition (440–497 °C): In this final stage, contributing 15.87% to the overall mass loss, more stable compounds or organic residues undergo decomposition. The relatively high-temperature range indicates greater thermal stability (require higher temperatures for breakdown).

The presence of AuNPs appears to influence the thermal stability of the composite system. Their incorporation appears to enhance thermal stability, potentially due to their unique properties, which may alter the decomposition pathway of the organic components. This effect might lead to a modification in the thermal profile observed during the analysis. Overall, the results emphasize the complex thermal behavior of the MEC-AuNPs system, highlighting the dynamic interactions between the NPs and the organic components throughout the degradation process. This improvement may arise from the unique properties of AuNPs, which influence the decomposition pathways of organic materials. Consequently, this interaction likely modifies the thermal profile observed during the analysis.

### 3.7. TPC and Evaluation of Antioxidant Potential

**Figure 11:**
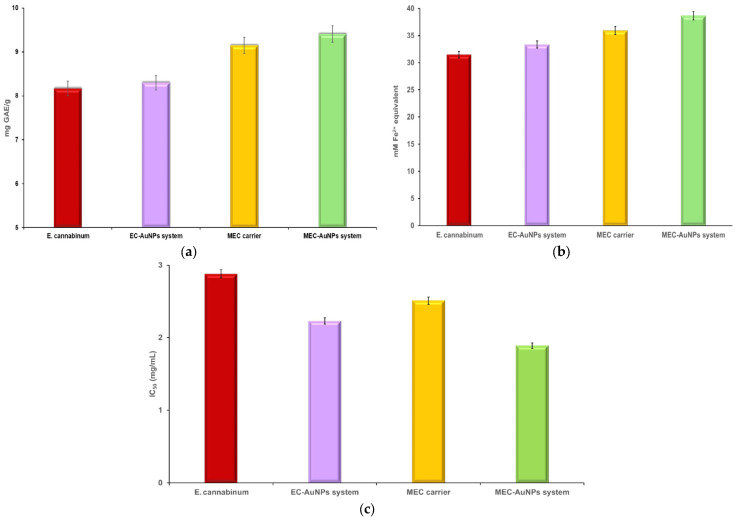
Schematic representation of TPC (**a**), FRAP (**b**), and DPPH (**c**) assays results for *E. cannabinum*, EC-AuNPs system, MEC carrier, and MEC-AuNPs system. DPPH, 2,2-Diphenyl-1-picrylhydrazyl; FRAP, ferric reducing antioxidant power; TPC, total phenolic content.

Two specific in vitro assays, particularly FRAP and DPPH, were utilized to thoroughly evaluate the antioxidant capacity of *E. cannabinum*, the EC-AuNPs system, the MEC carrier, and the MEC-AuNPs system. These assays measure antioxidant potential by assessing the samples’ ability to reduce ferric ions (FRAP) and scavenge free radicals (DPPH). Additionally, a TPC assay was conducted to quantify the phenolic compounds present in each sample both before and after their encapsulation in the biopolymeric matrix, offering deeper insights into any changes in antioxidant properties. The results of these analyses are illustrated in the accompanying Table S1 and Figure 11a–c.

No significant differences were observed in the TPC or FRAP assays between *E. cannabinum* and the newly developed EC-AuNPs system. The results from the DPPH assay for *E. cannabinum* were consistent with values documented in the relevant literature [94].

However, the EC-AuNPs system demonstrated a notable increase of 22.5% in antioxidant activity in the DPPH assay when compared to *E. cannabinum*. This enhancement can be attributed to the capacity of AuNPs to donate electrons or hydrogen ions, which neutralizes DPPH free radicals, as well as modifications in the surface electric charge of the metallic NPs embedded within the plant matrix, and the synergistic interactions between AuNPs and bioactive phytoconstituents [47,95,96].

In contrast, the encapsulated samples, specifically the MEC carrier and MEC-AuNPs system, exhibited enhanced polyphenol content and antioxidant activity. The TPC assay indicated an increase of 12% for the MEC carrier and 13.36% for the MEC-AuNPs system, which can be ascribed to the protective effects of maltodextrin matrix as reported in the literature [97,98].

Similarly, the FRAP assay reflected increases in antioxidant activity of 14.2% and 15.88% for the MEC carrier and MEC-AuNPs system, respectively, compared to their non-encapsulated counterparts. The DPPH assay further demonstrated improvements in antioxidant activity of 12.84% for the MEC carrier and 15.24% for the MEC-AuNPs system following encapsulation. These findings highlight the significant role of encapsulation in providing structural stability and protecting bioactive compounds, resulting in enhanced antioxidant activity across multiple assays. The use of maltodextrin as an encapsulating agent not only safeguards the phytoconstituents but also contributes to the sustained antioxidant potential of the encapsulated systems.

### 3.8. Antimicrobial Screening

The antibacterial properties of *E. cannabinum* and the newly developed EC-AuNPs system were systematically evaluated following their encapsulation within a maltodextrin matrix. The assessment included measuring the diameters of inhibition zones (IZs) and comparing the results to citrate-coated AuNPs, as well as positive (Gentamicin) and negative (DMSO) controls.

Antimicrobial efficacy was tested against a diverse range of pathogenic microorganisms, including *S. aureus* (Gram-positive), *B. subtilis* (Gram-positive), *B. cereus* (Gram-positive), *P. aeruginosa* (Gram-negative), *E. coli* (Gram-negative), and *S. typhi* (Gram-negative), utilizing the agar well diffusion method. The results are presented in Table 7.

The summarized data in Table 7 indicate that both *E. cannabinum* and the EC-AuNPs system demonstrated significant antibacterial activity against all tested pathogens. This highlights the potential of the EC-AuNPs system as an effective antimicrobial agent, likely due to the synergistic effects of *E. cannabinum*’s bioactive compounds and the unique properties of AuNPs within the maltodextrin matrix.

The MEC-AuNPs system exhibited the highest inhibition antibacterial activity across all tested pathogens, with particularly notable results against *S. aureus*. At a concentration of 200 μg/mL, it achieved the highest IZ diameter (74.07 ± 0.16 mm), surpassing the MEC carrier (70.33 ± 0.41 mm), the EC-AuNPs system (71.04 ± 0.38 mm), and Gentamicin (9.58 ± 0.51 mm). In contrast, citrate-coated AuNPs demonstrated minimal efficacy, with a maximum IZ of just 8.05 mm.

Similar trends were observed against *B. subtilis*, where the MEC-AuNPs system again recorded the largest IZ (74.79 mm), exceeding the MEC carrier (68.14 mm) and the EC-AuNPs system (72.57 mm), while citrate-coated AuNPs remained largely ineffective (8.85 mm). Against *B. cereus*, the MEC-AuNPs system maintained its superior performance with an IZ of 76.26 mm, compared to 72.03 mm for the MEC carrier and 74.14 mm for the EC-AuNPs system, while citrate-coated AuNPs exhibited limited activity (8.94 mm).

For *P. aeruginosa*, the MEC-AuNPs system produced the largest IZ (71.49 mm), slightly outperforming the MEC carrier (69.87 mm) and the EC-AuNPs system (69.27 mm). Citrate-coated AuNPs showed weak activity, with an IZ of 8.91 mm. Against *E. coli*, the MEC-AuNPs system again led with an IZ of 75.18 mm, followed by the EC-AuNPs system (73.63 mm) and the MEC carrier (70.94 mm). Citrate-coated AuNPs demonstrated moderate activity in this case, achieving an IZ of 22.08 mm.

The most dramatic results were observed against *S. typhi*, where the MEC-AuNPs system achieved an outstanding IZ of 96.24 mm, far exceeding the EC-AuNPs system (94.06 mm) and the MEC carrier (69.87 mm).

Notably, citrate-coated AuNPs displayed enhanced activity against this bacterium, achieving an IZ of 59.66 mm, likely due to its intrinsic sensitivity to AuNPs.

Overall, the MEC-AuNPs system consistently outperformed all other samples, demonstrating IZs three to eight times larger than those of Gentamicin, underscoring a synergistic effect between the maltodextrin matrix, bioactive phytoconstituents, and AuNPs.

Alternatively, *E. cannabinum* itself exhibited strong antibacterial properties, surpassing Gentamicin across all pathogens. Its activity was particularly pronounced against Gram-positive bacteria (*S. aureus, B. subtilis, B. cereus*), with IZs exceeding 67 mm, while for Gram-negative bacteria, although slightly smaller, the IZs were still significantly larger than those produced by Gentamicin [5,73,99]. These findings highlight the potential of the MEC-AuNPs system and *E. cannabinum* as effective antimicrobial agents.

To corroborate the antibacterial efficacy of all samples (*E. cannabinum,* citrate-coated AuNPs, the newly prepared EC-AuNPs system, MEC carrier, and MEC-AuNPs system), the MICs and MBCs were determined against all bacterial strains. The results are presented in Table 8.

All samples, especially the MEC-AuNPs and EC-AuNPs, showed lower MIC/MBC values against Gram-positive bacteria (*S. aureus*, *B. subtilis* and *B. cereus*) compared to Gram-negative bacteria (*P. aeruginosa*, *E. coli*, *S. typhi*). However, even against Gram-negative bacteria, the tested systems outperformed Gentamicin, showcasing their broad-spectrum efficacy.

The finding indicates that the MEC-AuNPs system consistently exhibited the lowest MIC and MBC values across all tested pathogens, indicating its superior antimicrobial potency. Moreover, their MIC and MBC values were frequently 2–4 times lower than Gentamicin, particularly against Gram-negative bacteria like *E. coli* and *S. typhi*.

Both the EC-AuNPs system and MEC carrier demonstrated better activity than *E. cannabinum.*

The EC-AuNPs system outperformed the MEC carrier in most cases but was generally slightly less effective than the MEC-AuNPs system.

While citrate-coated AuNPs showed moderate efficacy, their MIC/MBC values were higher than those of the functionalized systems (MEC-AuNPs and EC-AuNPs). This highlights the importance of biofunctionalization in enhancing antimicrobial activity. In summary, the MEC-AuNPs system is the most effective antimicrobial agent, with the lowest MICs and MBCs across all pathogens, outperforming Gentamicin and other test samples. This highlights the synergistic benefits of combining AuNPs with the maltodextrin carrier for enhanced bioactivity. The EC-AuNPs system also showed strong results, while citrate-coated AuNPs and *E. cannabinum* were less effective individually.

### 3.9. Cell Viability Assay

**Figure 12:**
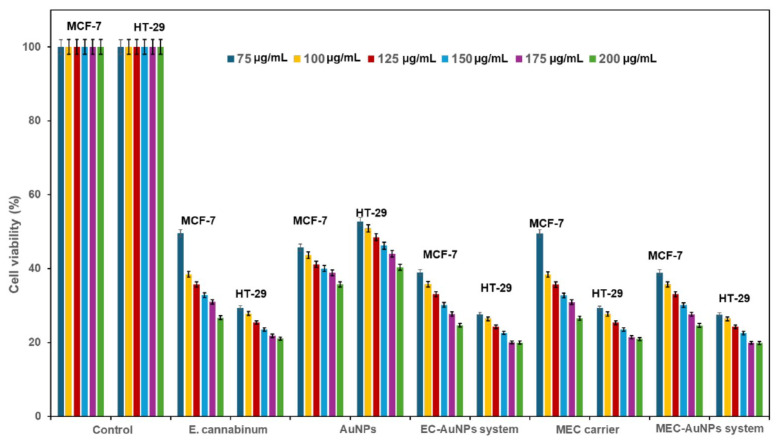
Viability of HT-29 and MCF-7 cells, 24 h after co-incubation with different concentrations of *E. cannabinum* sample, EC-AuNPs system, MEC carrier, and MEC-AuNPs system. Positive control wells contained untreated cells, MTT solution, and DMSO. Data are represented as mean ± SD (*n* = 3). DMSO, dimethyl sulfoxide; MTT, 3-(4,5-Dimethylthiazol-2-yl)-2,5 diphenyl tetrazolium bromide; SD, standard deviation.

Figure 12 delineates the results of cell viability assessments derived from MTT assays conducted on two cancer cell lines: MCF-7 (breast cancer) and HT-29 (colon cancer).

The study evaluates the cytotoxic effects exerted by *E. cannabinum*, AuNPs, the EC-AuNPs system, the MEC carrier, and the MEC-AuNPs system across a concentration range of 75–200 µg/mL. The viability percentages reflect the metabolic activity of viable cells, where enhanced viability suggests elevated mitochondrial function and diminished cytotoxic effects. Conversely, reduced cell viability indicates an increased cytotoxic activity from the test samples.

*E. cannabinum* exhibited moderate cytotoxicity, characterized by a dose-dependent decline in viability across both cell lines. Notably, MCF-7 cells displayed heightened sensitivity than HT-29, particularly at elevated concentrations (175–200 µg/mL). At the highest concentration tested (200 μg/mL), cell viability in MCF-7 fell below 50%, indicating a significant cytotoxic impact. In contrast, HT-29 cells maintained comparatively higher viability across all concentrations tested.

AuNPs demonstrated mild to moderate cytotoxicity towards both cancer cell lines, with a noted dose-dependent decrease in viability. Minimal cytotoxicity was observed at lower concentrations (75–125 µg/mL), with cell viability above 80%. However, at higher concentrations (175–200 µg/mL), a slight decrease in viability was recorded, though it remained above 60% for both cell lines, with a marginally greater cytotoxicity observed in MCF-7 cells.

Results from the MTT assay revealed that the EC-AuNPs system provoked a significant, dose-dependent decrease in cell viability, falling below 50% at concentrations exceeding 125 µg/mL for both cell lines. MCF-7 cells displayed marginally greater sensitivity compared to HT-29. The MEC carrier demonstrates moderate cytotoxicity, exhibiting a dose-dependent decline in cell viability, particularly pronounced at concentrations exceeding 150 µg/mL. At the maximum concentration of 200 µg/mL, the viability of MCF-7 cells approaches 50%, while that of HT-29 cells remains slightly higher. According to the results from the MTT assay, the MEC-AuNPs system demonstrates highly effective cytotoxicity against both MCF-7 and HT-29 cell lines, outperforming all other tested samples, which include *E. cannabinum*, the EC-AuNPs system, and the MEC carrier. At concentrations exceeding 150 µg/mL, cell viability declines to below 40% and drops to below 20% at 200 µg/mL for both cell lines. It is noteworthy that MCF-7 cells exhibit marginally lower viability compared to HT-29 cells, suggesting a greater sensitivity to this sample.

**Figure 13:**
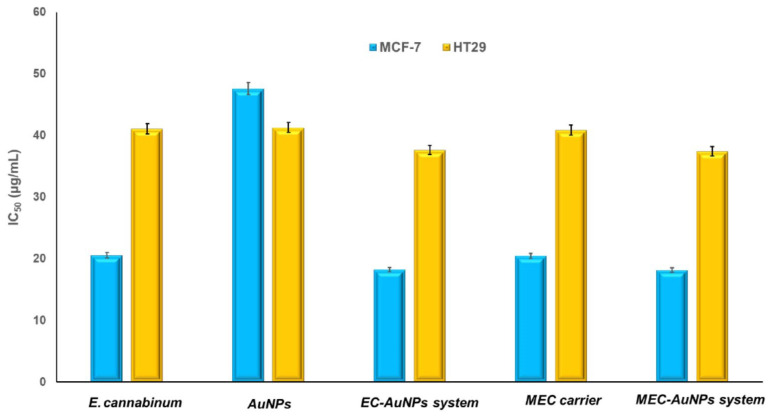
In vitro cytotoxicity of *E. cannabinum*, AuNPs, the EC-AuNPs system, the MEC carrier, and the MEC-AuNPs system, as a function of concentration against MCF-7 and HT-29 cells (after 24 h). Data are represented as mean ± SD (*n* = 3).

Figure 13 illustrates the IC_50_ values from in vitro cytotoxicity assays for *E. cannabinum*, AuNPs, the EC-AuNPs system, the MEC carrier, and the MEC-AuNPs system, highlighting their effectiveness in inhibiting cell viability.

For MCF-7 cells, the obtained IC_50_ values are as follows: the MEC-AuNPs system exhibits an IC_50_ of 18.19 ± 0.05 μg/mL, followed closely by the EC-AuNPs system at 18.22 ± 0.09 μg/mL.

The MEC carrier demonstrates an IC_50_ of 20.48 ± 0.11 μg/mL, while *E. cannabinum* has an IC_50_ of 20.58 ± 0.03 μg/mL. Citrate-coated AuNPs show the highest IC_50_ value at 47.62 ± 0.17 μg/mL.

In the case of HT-29 cells, the IC_50_ values recorded are 37.44 ± 0.068 μg/mL for the MEC-AuNPs system, 37.66 ± 0.034 μg/mL for the EC-AuNPs system, 40.89 ± 0.045 μg/mL for the MEC carrier, 41.09 ± 0.024 μg/mL for *E. cannabinum*, and 41.32 ± 0.039 μg/mL for citrate-coated AuNPs.

These results affirm that the MEC-AuNPs system exhibits the strongest cytotoxic effect, as indicated by the lowest IC_50_ values, followed closely by the EC-AuNPs system.

The MEC carrier and *E. cannabinum* demonstrate slightly lower efficacy compared to both the MEC and EC-AuNPs systems, yet their IC_50_ values remain lower than those of citrate-coated AuNPs. Additionally, the IC_50_ values for MCF-7 cells are consistently lower than those for HT-29 cells across all samples, suggesting that MCF-7 breast cancer cells are more sensitive to the tested samples than the HT-29 colon cancer cells.

## 4. Discussion

*E. cannabinum*, a renowned medicinal plant with deep roots in Romanian ethnomedicine, has recently garnered significant attention for its remarkable biological activities [5,18]. Amid the growing global challenge of AMR, there is an urgent need to reassess current therapeutic strategies for combating infectious diseases [24]. This has spurred a shift toward innovative approaches, emphasizing selective targeting strategies for advanced antimicrobial agents [24]. Medicinal plants like *E. cannabinum* are at the forefront of this shift, particularly as sources for novel, plant-based antibiotics enhanced by cutting-edge nanotechnology. Furthermore, recent research has unveiled the plant’s potent antitumoral properties across multiple cancer cell lines, emphasizing its dual potential as a transformative agent in infectious disease treatment and oncology.

### 4.1. Chemical Screening

The chemical composition of *E. cannabinum* was comprehensively analyzed, revealing a total of 80 phytoconstituents identified through GC–MS and ESI–QTOF–MS techniques. These compounds span a diverse spectrum of bioactive categories, including terpenoids, flavonoids, alkaloids, fatty acids, phytosterols, phenolic acids, esters, hydrocarbons, fatty alcohols, aldehydes, phenylpropanoids, phytoecdysteroids, and coumarins.

Terpenoids, constituting 25% of the total phytoconstituents in *E. cannabinum* (Figure 1), represent the main class of bioactive compounds identified. They exhibit a broad spectrum of therapeutic properties, including antitumor, antimicrobial, antiviral, analgesic, antispasmodic, anti-inflammatory, cardioprotective, antihyperglycemic, and immunomodulatory effects [100].

Flavonoids, the second class of metabolites, accounting for 16.3% of the plant’s phytochemicals (Table 2; Figure 1), are bioactive metabolites with notable biological activities, including antioxidant, antiviral, antimicrobial, antitumor, cardioprotective, and neuroprotective properties [101].

Alkaloids are the third class of biomolecules, comprised of 11.3% (Table 2) of the total phytochemicals identified in the *E. cannabinum* sample, and exhibit remarkable biological properties: antitumoral, antimicrobial, antifungal, antiviral, antidiabetic, anti-inflammatory, sedative, antihypertensive, antitussive, and antimitotic [102].

Fatty acids comprise 5% of total phytochemicals from the *E. cannabinum* sample, with two essential fatty acids, specifically one ω-3 acid (α-linolenic acid) and one ω-6 acid (linoleic acid), one saturated fatty acid (palmitic acid), and one monosaturated fatty acid (oleic acid) (Table 2). These compounds possess antioxidant, antimicrobial, anti-inflammatory, neuroprotective, and cardioprotective properties [103].

Phytosterols represent 5% of the total phytoconstituents (Table 2) and possess antioxidant, neuroprotective, cardioprotective, anti-inflammatory, antitumor, and immunomodulatory effects [104].

Phenolic acids show antioxidant, antibacterial, antitumor, anti-inflammatory, anti-allergic, antidiabetic, cardioprotective, and neuroprotective properties [105,106].

The phenylpropanoid estragole (Table 2) exhibits antioxidant, antiviral, antibacterial, anti-inflammatory, and immunomodulatory activity [107].

The phytoecdysteroid compound β-ecdysone (Table 2) acts as a neuroprotective, bone regeneration, antidiabetic, anti-obesity, and cytotoxic agent [108].

Coumarin (Table 2) presents antitumoral, anti-inflammatory, antibacterial, antifungal, neuroprotective, and anticoagulant properties [109].

### 4.2. Innovative Phytocarrier Systems with Antioxidant, Antimicrobial, and Cytotoxic Potential

The fusion of nanotechnology with engineered delivery systems employing metallic NPs offers a groundbreaking approach to addressing critical challenges in modern medicine, including bacterial resistance to antibiotics and therapeutic resistance in cancer.

Traditional phytoconstituents, despite their potent biological activity, are often hindered by poor stability and bioavailability, limiting their clinical potential. Advanced nanocarrier systems overcome these deficiencies by enhancing stability, enabling precise adsorption, and improving therapeutic outcomes. By integrating the unique properties of metallic NPs, these systems provide unparalleled benefits such as targeted activity, sustained and controlled drug release, reduced dosage requirements, and minimized toxicity [37,38,46,54,55,110]. Building on this concept, novel delivery systems were developed using *E. cannabinum*, leveraging advanced micro-spray encapsulation techniques. The first system involves the encapsulation of *E. cannabinum* phytoconstituents into maltodextrin microcapsules (MEC carrier), combining the biological activity of the plant’s phytoconstituents with the stabilizing and biocompatible properties of the maltodextrin biopolymer. The second system, the MEC-AuNPs system, integrates AuNPs within the maltodextrin matrix, enhancing the therapeutic potential by adding the unique biological activity of AuNPs. This dual approach represents an innovative strategy designed to tackle two of the most critical challenges in contemporary medicine: the mitigation of bacterial resistance to antibiotics and the counteraction of drug resistance in cancer cells. Moreover, this strategy enhances target specificity, facilitates controlled and sustained release of active compounds, and significantly boosts stability and permeability.

Understanding the thermal behavior of phytocarrier systems is critical for optimizing their design, stability, and performance in drug delivery and therapeutic applications. Thermal analysis provides valuable insights into the stability, decomposition, and interaction of the active phytoconstituents with the carrier matrix, which directly influences the efficacy and shelf life of the system. This study helps confirm the system’s structural integrity, assess its compatibility, and optimize controlled release properties under physiological conditions. By identifying decomposition patterns and thermal transitions, thermal behavior evaluation ensures that the system performs effectively during storage, processing, and application, making it an indispensable tool for designing robust and efficient phytocarrier systems.

A comparative analysis of thermal characteristics for *E. cannabinum* and the MEC carrier reveals that both samples exhibit considerable mass loss during the thermal degradation process; however, *E. cannabinum* demonstrates a total mass loss of 75.18%, which is significantly higher than that of the MEC carrier. Furthermore, both samples undergo multiple decomposition phases, although the temperature ranges and corresponding mass losses differ between these samples. The DTG peaks for both materials indicate critical points of thermal activity, albeit occurring at varying temperatures and stages. Both samples exhibit exothermic reactions during their decomposition phases, suggesting the release of energy as the structural integrity of the samples deteriorates. *E. cannabinum* displays a broader range of organic constituents that influence its thermal behavior, while the MEC carrier underscores the protective role of maltodextrin.

In summary, although herb and MEC carrier present complex thermal behaviors with specific temperature ranges for decomposition, the thermal profile of *E. cannabinum* is characterized by a greater mass loss and a more diverse array of organic compounds. In contrast, the MEC carrier emphasizes distinct phases predominantly shaped by maltodextrin and its thermal stability under stress.

Similarly, a comparative analysis of the thermal behavior of EC-AuNPs and MEC-AuNPs reveals that both systems demonstrate similar water loss during the initial stages of thermal treatment. However, the MEC-AuNPs formulation exhibits slightly superior moisture retention, which may be attributable to its specific composition and the interactions within the matrix. Regarding organic degradation, the MEC-AuNPs system is characterized by a broader temperature range for significant thermal decomposition, spanning from 205 °C to 390 °C, in contrast to the EC-AuNPs system, which experiences degradation primarily within the ranges of 181–244 °C and 245–354 °C. This observation suggests that the MEC-AuNPs undergo a more gradual and stable breakdown, likely facilitated by the protective effects of the maltodextrin matrix. Additionally, the thermal stability of the MEC-AuNPs system surpasses that of the EC-AuNPs system, with stable compounds degrading at elevated temperatures of 440–497 °C, compared to the EC-AuNPs, which degrades at temperatures ranging from 390–427 °C. Both systems exhibit stabilization effects resulting from the presence of AuNPs, although this effect is more pronounced in the MEC-AuNPs formulation, possibly due to the synergistic interactions between the AuNPs and the maltodextrin matrix. The data also indicate that the MEC-AuNPs system experiences greater total mass loss, quantified at 86.80%, compared to the EC-AuNPs system at 62.59%. This difference may reflect a higher organic content in the MEC-AuNPs system or the combined degradation of both the maltodextrin and encapsulated bioactive compounds. In conclusion, while both systems exhibit complex, multi-stage decomposition behaviors influenced by AuNPs, the MEC-AuNPs system demonstrates enhanced thermal stability, a more regulated decomposition process, and greater thermal resilience in the later stages of thermal analysis. The contributions of the maltodextrin matrix are likely essential in accounting for these observed differences, thereby enhancing the robustness of the MEC-AuNPs system under thermal stress.

A comprehensive evaluation of the antioxidant potential of herbal products requires multiple assays, ensuring accuracy and depth in the analysis. In vitro tests are especially valuable for examining samples with complex compositions of biomolecules, as they offer precise and controlled conditions to assess antioxidant activity. These assays provide critical insights into the synergistic effects of diverse bioactive compounds present in herbal formulations, allowing for a detailed understanding of their ability to neutralize free radicals and mitigate oxidative stress.

The antioxidant activity of *E. cannabium* is intricately associated with its highly active phytoconstituents [5,10,16,72]. In contrast, the antioxidant potential of the EC-AuNPs system arises from the synergistic effect of the phytochemicals and the conjugated AuNPs. The findings indicate that within the EC-AuNP system, the AuNPs, when combined with the phytoconstituents, may function as electron or hydrogen donors, serve as reducing agents, and quench singlet oxygen, thereby enhancing the overall antioxidant capacity [111].

Furthermore, the encapsulation techniques that employ maltodextrin in both the MEC carrier and MEC-AuNPs systems result in significant enhancements in TPC and antioxidant activity. This emphasizes the protective role of the maltodextrin matrix [112]. The findings indicate that encapsulation not only stabilizes bioactive compounds but also boosts their antioxidant potential, thereby underscoring the critical role of formulation strategies in enhancing the functional properties of plant-derived substances. Overall, this research highlights the promising application of AuNPs and encapsulation technologies for developing effective antioxidant formulations.

A comparative analysis of the EC-AuNPs and MEC-AuNPs systems indicates both are highly effective; however, the MEC-AuNPs system generally outperformed EC-AuNPs, likely attributed to the stabilizing and controlled release characteristics conferred by the maltodextrin matrix.

Citrate-coated AuNPs exhibited limited antimicrobial effectiveness, particularly against Gram-positive bacteria, and were less effective than Gentamicin in most cases, findings which align well with the existing literature [98,109], highlighting the critical role of functionalization in enhancing antimicrobial action. Both the EC-AuNPs system and the MEC carrier exhibited remarkable activity, surpassing Gentamicin across all the bacterial strains tested.

Comparative IZ diameter analysis suggests that the MEC carrier typically exhibited slightly greater inhibition than *E. cannabinum*, indicating that the maltodextrin matrix may enhance the stabilization and delivery of the bioactive compounds [5,73,113,114]. In summary, Gram-negative bacteria such as *E. coli* and *S. typhi* were inhibited more effectively by all samples when compared to *P. aeruginosa*, and Gram-positive strains (*S. aureus*, *B. subtilis*, and *B. cereus*) also exhibited pronounced inhibition, reflecting the broad-spectrum antimicrobial efficacy of the EC-AuNPs and MEC-AuNPs systems.

The EC-AuNPs system demonstrated high effectiveness, albeit slightly less potent than the MEC-AuNPs system, which exhibits the best overall performance against all pathogens, showcasing the advantages of enhanced stability, controlled release, and increased efficacy due to the maltodextrin matrix and AuNPs.

Conversely, the citrate-coated AuNPs revealed limited antimicrobial potential, underscoring the necessity of optimized functionalized delivery systems to maximize therapeutic activity.

In vitro cytotoxicity assays are essential tools for assessing the potential toxicity of specific compounds using cell culture models. These assays provide insights into how a compound affects cell viability, growth, morphology, and metabolism, as well as its ability to inhibit cell proliferation. Such evaluations are crucial for understanding cytotoxicity and serve as a foundational step in assessing the bioavailability of compounds. Among the various methodologies employed, colorimetric assays, particularly the MTT assay, are widely favored for their cost-effectiveness and reliability in measuring cell viability in vitro [115].

According to the test results, the observed moderate cytotoxicity attributable to *E. cannabinum* can be linked to its phytochemical constituents, including flavonoids, tannins, terpenes, and polyphenols, which are known to induce oxidative stress, disrupt cellular membranes, and inhibit the proliferation of cancer cells. The increased sensitivity of MCF-7 cells compared to HT-29 is likely due to differential metabolic pathways that modulate breast cancer cell survival and their susceptibility to oxidative damage [5,7,10,18,19,21,23,77].

The cytotoxicity associated with AuNPs is attributed to the ROS generation and their interaction with cellular proteins and DNA, disrupting essential cellular processes, but insufficient to cause considerable cell death. The limited efficacy could be ascribed to the absence of functionalization, which diminishes specificity and potency against cancer cells [116].

The EC-AuNPs system showcased enhanced cytotoxicity relative to citrate-coated AuNPs, attributed to the synergistic interaction between AuNPs and the herb components. Phytochemicals from *E. cannabinum* disrupt cancer cell membranes and inhibit survival pathways, while AuNPs enhance these effects through ROS generation and interactions with DNA and proteins, consequently impairing mitochondrial function and triggering apoptotic pathways. The more pronounced effect on MCF-7 underscores the higher sensitivity of this cell line to the biofunctionalized system, suggesting that the functionalized approach enhances cellular uptake and effectively targets cancer-specific pathways.

These findings indicate that the maltodextrin matrix functions effectively as a controlled delivery system for cytotoxic compounds that disrupt vital metabolic processes in cancer cells. Although the cytotoxicity is moderate, the MEC carrier acts as an efficient platform, significantly enhancing the bioavailability of the incorporated bioactive compounds.

The observed results may be attributed to the synergistic interactions between the maltodextrin matrix, the phytoconstituents, and the AuNPs. The mechanisms responsible for this enhanced efficacy may involve the generation of ROS, cellular membrane disruption, and interference with critical cancer cell signaling pathways. These effects are amplified by the improved delivery, sustained release, and stability of the functionalized AuNPs. In conclusion, the MEC-AuNPs system was the most effective in inducing cytotoxicity, followed by the EC-AuNPs system. This highlights the critical role of combining functionalized NPs with biomolecules and incorporation in a biopolymeric matrix to enhance anticancer activity.

Both the EC-AuNPs and MEC-AuNPs systems exhibit pronounced, dose-dependent effects, with a particular susceptibility observed in MCF-7 cells. In contrast, *E. cannabinum*, citrate-coated AuNPs, and the MEC carrier show moderate effects, especially toward HT-29 cells, underscoring the superior potential of functionalized NPs with bioactive compounds in targeted cancer therapy.

## 5. Conclusions

This study highlights the innovative development of a plant-based system incorporating AuNPs into the herbal matrix of *E. cannabinum*, resulting in the formation of the EC-AuNPs system. The successful integration of AuNPs was confirmed through a range of analytical techniques including FTIR spectroscopy, SEM, XRD, and DLS.

The research further explored the micro-spray encapsulation of both the *E. cannabinum* plant sample and the newly developed phytocarrier system (EC-AuNPs) within a maltodextrin matrix. This process led to the creation of two novel phytocarrier systems: the MEC carrier and the MEC-AuNPs. The characterization of these systems was validated through comprehensive analyses using the same techniques (FTIR, SEM–EDX, XRD, and DLS), confirming their structural and functional integrity.

Thermal stability assessments indicated that these systems maintain their properties under varying temperature conditions, which is crucial for their potential application. The study also included biological evaluations, such as antioxidant screening, antimicrobial assays, and in vitro cell viability tests. The results demonstrated that the EC-AuNPs and MEC-AuNPs systems possess significantly enhanced biological properties compared to *E. cannabinum* alone.

Overall, the findings of this research underscore the potential of the EC-AuNPs and MEC-AuNPs systems in various medical applications, presenting a promising avenue for the advancement of therapeutic solutions through the integration of nanotechnology with herbal medicine.

## Figures and Tables

**Table 1:** Main biomolecules identified by GC–MS analysis of *E. cannabinum* sample.

No.	t_R_ (min)	RI	Kováts RI	Compound	Formula	MW (g/mol)	Area (%)	Ref.
1	3.76	822	829	2-hexenal	C_6_H_10_O	98.14	1.24	[55,61]
2	5.78	928	901	heptanal	C_7_H_14_O	114.19	2.19	[62]
3	7.02	1022	1026	*p*-cymene	C_10_H_14_	134.22	0.89	[55,63]
4	10.89	957	954	benzaldehyde	C_7_H_6_O	106.12	0.92	[62]
5	11.37	1979	1973	palmitic acid	C_16_H_32_O_2_	256.42	1.89	[54]
6	12.76	1985	1089	terpinolene	C_10_H_16_	136.23	0.91	[64]
7	14.92	1321	1113	linalool	C_10_H_18_O	154.25	2.18	[65]
8	16.53	1599	1601	hexadecane	C_16_H_34_	226.44	1.22	[54]
9	17.66	1109	1095	nonanal	C_9_H_18_O	142.24	6.67	[66]
10	22.27	1795	1743	chamazulene	C_14_H_16_	184.28	11.53	[67]
11	24.82	1197	1199	decanal	C_10_H_20_O	156.26	2.21	[66]
12	27.75	1921	1918	thymol	C_10_H_14_O	150.22	3.35	[65]
13	29.86	2077	2080	phytol	C_20_H_40_O	296.50	2.89	[54]
14	31.55	1551	1553	8,9-dehydrothymol	C_10_H_12_O	148.20	2.67	[68]
15	32.74	1243	1245	methyl thymol	C_11_H_16_O	164.24	2.85	[64]
16	34.48	1579	1578	spathulenol	C_15_H_24_O	220.35	1.95	[55,63]
17	35.93	1803	1805	octadecane	C_18_H_38_	254.50	6.58	[62]
18	37.69	1899	1901	nonadecane	C_19_H_40_	268.50	19.72	[62]
19	39.57	3292	3294	β-sitosterol	C_29_H_50_O	414.70	0.97	[54]
20	41.27	1295	1497	germacrene D	C_15_H_24_	204.35	1.96	[55,63]
21	43.17	2695	2999	eicosane	C_20_H_42_	282.50	2.05	[69]
22	45.14	1363	1364	neryl acetate	C_12_H_20_O_2_	196.29	1.53	[70]
23	47.84	1355	1531	cadinene	C_15_H_26_	204.35	0.95	[55]
24	61.75	1625	1627	eudesmol	C_15_H_26_O	222.37	3.33	[71]

GC–MS, gas chromatography–mass spectrometry; MW, molecular weight; RI, retention index; t_R_, retention time.

**Table 2:** Phytochemicals identified by mass spectrometry analysis in *E. cannabinum* sample.

No.	*m*/*z* Detected	Theoretic *m*/*z*	Formula	Tentative of Identification	Category	Ref.
1	147.15	146.14	C_9_H_6_O_2_	coumarin	coumarins	[10]
2	257.43	256.42	C_16_H_32_O_2_	palmitic acid	fatty acids	[5,76]
3	279.39	278.40	C_18_H_30_O_2_	α-linolenic acid	fatty acids	[76]
4	281.39	280.40	C_18_H_32_O_2_	linoleic acid	fatty acids	[76]
5	283.51	282.50	C_18_H_34_O_2_	oleic acid	fatty acids	[76]
6	287.23	286.24	C_15_H_10_O_6_	kaempferol	flavonoids	[5,17]
7	303.24	302.23	C_15_H_10_O_7_	quercetin	flavonoids	[5,18]
8	315.29	314.29	C_17_H_14_O_6_	pectolinaringenin	flavonoids	[17]
9	331.28	330.29	C_17_H_14_O_7_	jaceosidin	flavonoids	[17]
10	333.27	332.26	C_16_H_12_O_8_	patuletin	flavonoids	[76]
11	345.29	344.30	C_18_H_16_O_7_	eupatilin	flavonoids	[10,17]
12	361.29	360.30	C_18_H_16_O_8_	centaureidin	flavonoids	[17]
13	345.29	344.30	C_18_H_16_O_7_	eupatorin	flavonoids	[5,10]
14	301.27	300.26	C_16_H_12_O_6_	hyspidulin	flavonoids	[5,17]
15	449.41	448.40	C_21_H_20_O_11_	astragalin	flavonoids	[5,17]
16	465.39	464.40	C_21_H_20_O_12_	hyperoside	flavonoids	[5,17]
17	595.51	594.50	C_27_H_30_O_15_	nicotiflorin	flavonoids	[76]
18	611.49	610.50	C_27_H_30_O_16_	rutin	flavonoids	[17,18]
19	181.17	180.16	C_9_H_8_O_4_	caffeic acid	phenolic acids	[18]
20	355.31	354.31	C_16_H_18_O_9_	chlorogenic acid	phenolic acids	[17]
21	101.13	100.12	C_5_H_8_O_2_	angelic acid	alkaloids	[72]
22	142.22	141.21	C_8_H_15_NO	trachelanthamidine	alkaloids	[72]
23	156.20	155.19	C_8_H_13_NO_2_	trachelantic acid	alkaloids	[72]
24	157.21	157.21	C_8_H_15_NO_2_	turneforcidine	alkaloids	[73]
25	163.19	162.18	C_7_H_14_O_4_	viridifloric acid	alkaloids	[72]
26	283.37	283.36	C_15_H_25_NO_4_	supinine	alkaloids	[72]
27	286.37	285.38	C_15_H_27_NO_4_	lindefoline	alkaloids	[72]
28	289.33	288.34	C_17_H_20_O_4_	9-acetoxy-8,10-epoxythymol 3-O-tiglate	alkaloids	[10]
29	300.37	299.36	C_15_H_25_NO_5_	rinderine	alkaloids	[72]
30	401.71	400.70	C_28_H_48_O	campesterol	sterols	[76]
31	413.69	412.70	C_29_H_48_O	stigmasterol	sterols	[10]
32	427.69	426.70	C_30_H_50_O	taraxasterol	sterols	[10]
33	415.71	414.70	C_29_H_50_O	β-sitosterol	sterols	[10]
34	135.23	134.22	C_10_H_14_	*p*-cymene	terpenes	[10,73]
35	137.24	136.23	C_10_H_16_	terpinolene	terpenes	[5,73,74]
36	149.21	148.20	C_10_H_12_O	8,9-dehydrothymol	terpenes	[10]
37	151.23	150.22	C_10_H_14_O	thymol	terpenes	[5,10,73]
38	155.25	154.25	C_10_H_18_O	linalool	terpenes	[5,73]
39	165.23	164.24	C_11_H_16_O	methyl thymol	terpenes	[10,16,73]
40	185.27	184.27	C_11_H_20_O_2_	hexyl tiglate	terpenes	[73]
41	185.29	184.28	C_14_H_16_	chamazulene	terpenes	[5,74]
42	193.31	192.30	C_13_H_20_O	beta-ionone	terpenes	[10]
43	197.28	196.29	C_12_H_20_O_2_	neryl acetate	terpenes	[16,73]
44	201.22	200.23	C_13_H_12_O_2_	dehydrotremetone	terpenes	[72]
45	203.24	202.25	C_13_H_14_O_2_	tremetone	terpenes	[72]
46	205.36	204.35	C_15_H_24_	germacrene D	terpenes	[10,16,73,74]
47	207.36	206.37	C_15_H_26_	cadinene	terpenes	[5]
48	221.34	220.35	C_15_H_24_O	spathulenol	terpenes	[16,74]
49	223.26	222.37	C_15_H_26_O	eudesmol	terpenes	[5,10]
50	249.33	248.32	C_15_H_20_O_3_	eupatolide	terpenes	[77]
51	297.51	296.50	C_20_H_40_O	phytol	terpenes	[5,74]
52	363.39	362.40	C_20_H_26_O_6_	eupatoriopicrin	terpenes	[77]
53	427.71	426.70	C_30_H_50_O	α-amyrin	terpenes	[76]
54	465.59	464.60	C_27_H_44_O_6_	β-ecdysone	phytoecdysteroids	[10]
55	149.19	148.20	C_10_H_12_O	estragole	phenylpropanoids	[72]
56	243.43	242.44	C_16_H_34_O	cetyl alcohol	fatty alcohols	[10]
57	271.51	270.50	C_18_H_38_O	stearyl alcohol	fatty alcohols	[74,76]
58	327.59	326.60	C_22_H_46_O	*n*-docosanol	fatty alcohols	[76]
59	299.49	298.50	C_20_H_42_O	arachidyl alcohol	fatty alcohols	[76]
60	227.43	226.44	C_16_H_34_	hexadecane	hydrocarbons	[5,73]
61	255.51	254.50	C_18_H_38_	octadecane	hydrocarbons	[73]
62	269.49	268.50	C_19_H_40_	*n*-nonadecane	hydrocarbons	[74]
63	283.51	282.50	C_20_H_42_	*n*-eicosane	hydrocarbons	[10]
64	297.61	296.60	C_21_H_44_	*n*-heneicosane	hydrocarbons	[10]
65	311.59	310.60	C_22_H_46_	*n*-docosane	hydrocarbons	[10]
66	49.08	46.07	C_2_H_6_O	dimethyl ether	miscellaneous	[76]
67	118.14	117.15	C_8_H_7_N	indole	miscellaneous	[73]
68	123.15	122.16	C_8_H_10_O	2-phenylethanol	miscellaneous	[5,73]
69	139.22	138.21	C_9_H_14_O	2-pentylfuran	miscellaneous	[73]
70	153.24	152.23	C_10_H_16_O	2-hexylfuran	miscellaneous	[5]
71	165.21	164.20	C_10_H_12_O_2_	thymoquinone	miscellaneous	[10]
72	218.26	218.25	C_13_H_14_O_3_	hydroxytremetone	miscellaneous	[76]
73	225.33	224.34	C_14_H_24_O_2_	bornyl isobutanoate	esters	[10]
74	239.38	238.37	C_15_H_26_O_2_	neryl isovalerate	esters	[10]
75	284.37	283.36	C_15_H_25_NO_4_	amabiline	esters	[75]
76	99.15	98.14	C_6_H_10_O	2-hexanal	aldehydes	[73]
77	107.13	106.12	C_7_H_6_O	benzaldehyde	aldehydes	[5,10,73]
78	121.14	120.15	C_8_H_8_O	phenylacetaldehyde	aldehydes	[73]
79	143.25	142.24	C_9_H_18_O	nonanal	aldehydes	[10]
80	157.25	156.26	C_10_H_20_O	decanal	aldehydes	[10]

**Table 3:** VOCs identified through MS in the *E. cannabinum* sample.

VOC Name	Odor
bornyl isobutanoate	camphoraceous
neryl isovalerate	apple
benzaldehyde	bitter almond
2-hexenal	green
phenylacetaldehyde	green
nonanal	rose
decanal	orange
carvone	minty
limonene	citric
phytol	floral
thymol	herbaceous
chamazulene	apples
β-ionone	floral
linalool	floral
β-caryophyllene	woody
*p*-cymene	woody
terpinolene	pine
neryl acetate	fruity
germacrene D	woody
cadinene	woody
spathulenol	earthy
eudesmol	spicy
2-phenylethanol	rose
indole	fecal
thymoquinone	pencil
cetyl alcohol	waxy
hexyl tiglate	green
methyl thymol	woody

MS, mass spectrometry; VOC, volatile organic compound.

**Table 4:** Characteristic vibrational peaks associated with phytochemicals from *E cannabinum* sample.

Phytochemicals Category	Wavenumber (cm^−1^)	Ref.
terpenoids	2348, 1738, 1088, 812	[78]
alkaloids	1637, 1600, 1404, 740, 665	[78]
coumarins	3429, 1728, 1609, 1267, 1135, 1255, 902–601	[79]
flavonoids	3402–3102, 1524, 1462, 1436, 1366, 1272	[55,80]
phenolic acids	1366, 1253, 1242, 1169–1102, 1032	[78,80]
fatty acids	2922, 1349, 1249, 1091, 745, 722	[47]
phytosterols	1463, 1380, 1060, 740	[81]
phenylpropanoids	3188, 3002, 1636, 1504, 1449, 1248	[82]
phytoecdysteroids	3305, 1655	[83,84]

**Table 5:** Particle diameter distribution of MEC carrier and MEC-AuNPs system microcapsules.

Sample	Particle Size Diameter (μm)		Volume Diameter (μm)		
	D[3,2]	D[4,3]	*d*_10_	*d*_50_	*d*_90_
MEC carrier	0.298 ± 0.002	0.380 ± 0.003	0.121 ± 0.001	0.312 ± 0.002	0.714 ± 0.006
MEC-AuNPs system	0.521 ± 0.004	0.649 ± 0.006	0.186 ± 0.001	0.689 ± 0.005	1.123 ± 0.013

D[3,2], surface-weighted mean diameter; D[4,3], volume-weighted mean diameter. The d_10_, d_50_, and d_90_ correspond to cumulative distributions at 10%, 50%, and 90%, respectively. AuNPs, gold nanoparticles; MEC, maltodextrin–*E. cannabinum* (EC).

**Table 6:** Encapsulation parameters for prepared carriers.

Sample Name	EE%	EC%	EY%
MEC carrier	61.12 ± 0.11	59.39 ± 0.26	60.55 ± 0.41
MEC-AuNPs system	61.85 ± 0.12	61.11 ± 0.36	61.27 ± 0.48

EC%, loading capacity; EE%, encapsulation efficiency; EY%, encapsulation yield.

**Table 7:** Antibacterial activity results against selected pathogenic microorganisms.

Pathogenic Microorganism	Sample	Inhibition Zone Diameter (mm)						
		Sample Concentration (μg/mL)					Positive Control (Gentamicin 100 μg/mL)	Negative Control (DMSO)
		100	125	150	175	200		
*Staphylococcus * *aureus*	*E. cannabinum*	37.13 ± 0.54	45.12 ± 0.87	56.33 ± 0.72	61.27 ± 0.41	67.16 ± 0.52	9.58 ± 0.51	0
	EC-AuNPs system	45.38 ± 0.25	51.06 ± 0.38	60.24 ± 0.29	69.73 ± 0.41	71.04 ± 0.38		
	citrate-coated AuNPs	3.05 ± 0.21	4.82 ± 0.32	6.33 ± 0.18	7.24 ± 0.62	8.05 ± 0.62		
	MEC carrier	39.49 ± 0.27	47.32 ± 0.45	59.46 ± 0.34	65.22 ± 0.11	70.33 ± 0.41		
	MEC-AuNPs system	48.62 ± 0.62	57.19 ± 0.36	62.53 ± 0.25	71.15 ± 0.42	74.07 ± 0.16		
*Bacillus subtilis*	*E. cannabinum*	35.03 ± 0.13	46.25 ± 0.33	57.81 ± 0.44	63.26 ± 0.16	68.14 ± 0.22	17.91 ± 0.17	0
	EC-AuNPs system	46.78 ± 0.32	53.02 ± 0.41	61.56 ± 0.35	70.68 ± 0.52	72.57 ± 0.27		
	citrate-coated AuNPs	3.13 ± 0.33	4.98 ± 0.45	6.78 ± 0.42	7.47 ± 0.22	8.85 ± 0.37		
	MEC carrier	36.11 ± 0.23	46.25 ± 0.33	57.81 ± 0.44	63.26 ± 0.16	68.14 ± 0.22		
	MEC-AuNPs system	48.17 ± 0.34	54.87 ± 0.41	62.08 ± 0.23	72.32 ± 0.51	74.79 ± 0.33		
*Bacillus cereus*	*E. cannabinum*	36.17 ± 0.44	47.81 ± 0.19	57.83 ± 0.32	64.06 ± 0.23	69.76 ± 0.63	28.64 ± 09	0
	EC-AuNPs system	48.03 ± 0.27	55.31 ± 0.15	63.41 ± 0.43	73.02 ± 0.33	74.14 ± 0.33		
	citrate-coated AuNPs	3.49 ± 0.81	4.83 ± 0.26	6.75 ± 0.17	7.49 ± 0.31	8.94 ± 0.14		
	MEC carrier	38.03 ± 0.26	48.42 ± 0.18	59.34 ± 0.47	66.65 ± 0.62	72.03 ± 0.38		
	MEC-AuNPs system	50.01 ± 0.71	57.14 ± 0.36	65.22 ± 0.47	74.92 ± 0.19	76.26 ± 0.51		
*Pseudomonas * *aeruginosa*	*E. cannabinum*	33.42 ± 0.37	41.21 ± 0.52	50.84 ± 0.35	61.36 ± 0.27	66.94 ± 0.45	18.64 ± 0.13	0
	EC-AuNPs system	39.92 ± 0.43	45.81 ± 0.21	54.16 ± 0.46	65.74 ± 0.16	69.27 ± 0.53		
	citrate-coated AuNPs	3.51 ± 0.11	4.86 ± 0.32	6.71 ± 0.27	7.51 ± 0.43	8.91 ± 0.58		
	MEC carrier	36.55 ± 0.25	44.74 ± 0.19	53.75 ± 0.41	64.86 ± 0.17	69.87 ± 0.53		
	MEC-AuNPs system	41.33 ± 0.63	47.46 ± 0.33	55.48 ± 0.21	67.33 ± 0.42	71.49 ± 0.17		
*Escherichia coli*	*E. cannabinum*	35.49 ± 0.72	46.65 ± 0.63	56.48 ± 0.27	63.82 ± 0.36	69.12 ± 0.51	20.71 ± 0.28	0
	EC-AuNPs system	47.21 ± 0.15	54.72 ± 0.46	62.58 ± 0.32	72.74 ± 0.29	73.63 ± 0.31		
	citrate-coated AuNPs	12.41 ± 0.22	15.15 ± 0.41	17.84 ± 0.53	19.94 ± 0.37	22.08 ± 0.25		
	MEC carrier	37.21 ± 0.53	48.52 ± 0.34	58.17 ± 0.41	65.35 ± 0.62	70.94 ± 0.19		
	MEC-AuNPs system	48.98 ± 0.34	56.07 ± 0.21	63.75 ± 0.18	74.09 ± 0.11	75.18 ± 0.45		
*Salmonella typhi*	*E. cannabinum*	34.22 ± 0.31	45.78 ± 0.15	55.32 ± 0.23	62.44 ± 0.42	68.54 ± 0.47	12.09 ± 38	0
	EC-AuNPs system	59.93 ± 0.22	66.48 ± 0.17	80.25 ± 0.34	89.88 ± 0.56	94.06 ± 0.17		
	citrate-coated AuNPs	23.88 ± 0.22	39.03 ± 0.16	46.27 ± 0.35	52.18 ± 0.42	59.66 ± 0.17		
	MEC carrier	38.07 ± 0.35	47.11 ± 0.22	56.78 ± 0.44	64.11 ± 0.18	69.87 ± 0.53		
	MEC-AuNPs system	62.53 ± 0.41	68.22 ± 0.34	82.07 ± 0.15	91.77 ± 0.41	96.24 ± 0.62		

**Table 8:** MICs and MBCs of samples against selected pathogenic microorganisms.

Pathogenic Microorganism	Sample	MIC (μg/mL)	MBC (μg/mL)	Gentamicin	
				MIC (μg/mL)	MBC (μg/mL)
*Staphylococcus aureus*	*E. cannabinum*	0.29 ± 0.12	0.30 ± 0.23	0.61 ± 0.56	0.61 ± 0.56
	citrate-coated AuNPs	0.42 ± 0.17	0.41 ± 0.09		
	EC-AuNPs system	0.21 ± 0.03	0.20 ± 0.16		
	MEC carrier	0.23 ± 0.22	0.22 ± 0.18		
	MEC-AuNPs system	0.19 ± 0.22	0.18 ± 0.18		
*Bacillus subtilis*	*E. cannabinum*	0.33 ± 0.16	0.32 ± 0.14	0.48 ± 0.21	0.44 ± 0.17
	citrate-coated AuNPs	0.43 ± 0.04	0.42 ± 0.08		
	EC-AuNPs system	0.26 ± 0.13	0.24 ± 0.09		
	MEC carrier	0.31 ± 0.18	0.30 ± 0.23		
	MEC-AuNPs system	0.28 ± 0.11	0.27 ± 0.15		
*Pseudomonas aeruginosa*	*E. cannabinum*	0.83 ± 0.17	0.81 ± 0.11	1.28 ± 0.02	1.27 ± 0.06
	citrate-coated AuNPs	0.74 ± 0.31	0.73 ± 0.29		
	EC-AuNPs system	0.64 ± 0.22	0.62 ± 0.17		
	MEC carrier	0.79 ± 0.13	0.78 ± 0.07		
	MEC-AuNPs system	0.61 ± 0.17	0.60 ± 0.09		
*Bacillus cereus*	*E. cannabinum*	0.98 ± 0.11	0.99 ± 0.14	1.98 ± 0.34	1.97 ± 0.27
	citrate-coated AuNPs	0.73 ± 0.23	0.72 ± 0.21		
	EC-AuNPs system	0.52 ± 0.07	0.59 ± 0.37		
	MEC carrier	0.87 ± 0.04	0.86 ± 0.07		
	MEC-AuNPs system	0.48 ± 0.14	0.47 ± 0.21		
*Escherichia coli*	*E. cannabinum*	0.68 ± 0.05	0.65 ± 0.18	0.82 ± 0.19	0.82 ± 0.17
	citrate-coated AuNPs	0.42 ± 0.14	0.41 ± 0.11		
	EC-AuNPs system	0.38 ± 0.09	0.37 ± 0.16		
	MEC carrier	0.64 ± 0.22	0.62 ± 0.27		
	MEC-AuNPs system	0.28 ± 0.05	0.28 ± 0.07		
*Salmonella typhi*	*E. cannabinum*	0.62 ± 0.14	0.62 ± 0.12	1.12 ± 0.26	1.11 ± 0.31
	citrate-coated AuNPs	0.38 ± 0.21	0.37 ± 0.17		
	EC-AuNPs system	0.33 ± 0.23	0.32 ± 0.19		
	MEC carrier	0.58 ± 0.11	0.57 ± 0.08		
	MEC-AuNPs system	0.24 ± 0.09	0.23 ± 0.04		

Values are expressed as the mean ± SD (*n* = 3). MBC, minimum bactericidal concentration; MIC, minimum inhibitory concentration; SD, standard deviation.

## Data Availability

The original contributions presented in this study are included in the article. Further inquiries can be directed to the corresponding author.

## Supplementary Materials

The following supporting information can be downloaded at https://www.mdpi.com/article/10.3390/polym17040482/s1: Figure S1, Total ion chromatogram of *E. cannabinum* sample; Figure S2, Mass spectrum of *E. cannabinum* sample; Table S1, Results of TPC and selected antioxidant assays for samples before and after encapsulation.
