# Supplementary material for: Wild-Grown Romanian Eupatorium cannabinum: Advancing Phyto-Nanocarriers via Maltodextrin Micro-Spray Encapsulation—Metabolite Profiling, Antioxidant, Antimicrobial, and Cytotoxicity Insights
Source: Polymers (Basel). 2025 Feb 12;17(4):482. doi: 10.3390/polym17040482 (PMC11858835; doi:10.3390/polym17040482)
Supplement: Supplementary file 1 [file polymers-17-00482-s001.zip › polymers-3409023-supplementary.pdf]

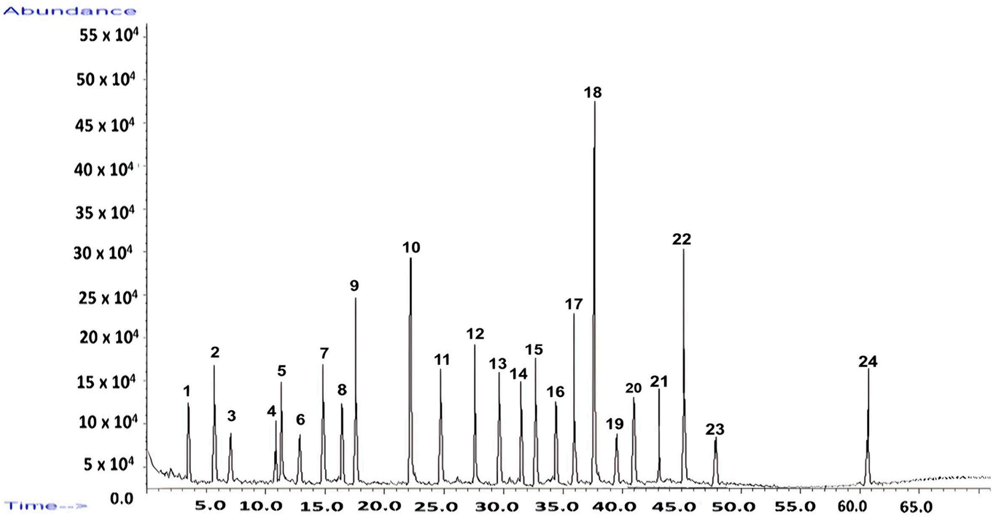

Figure S1. Total ion chromatogram of *E. cannabinum* sample.

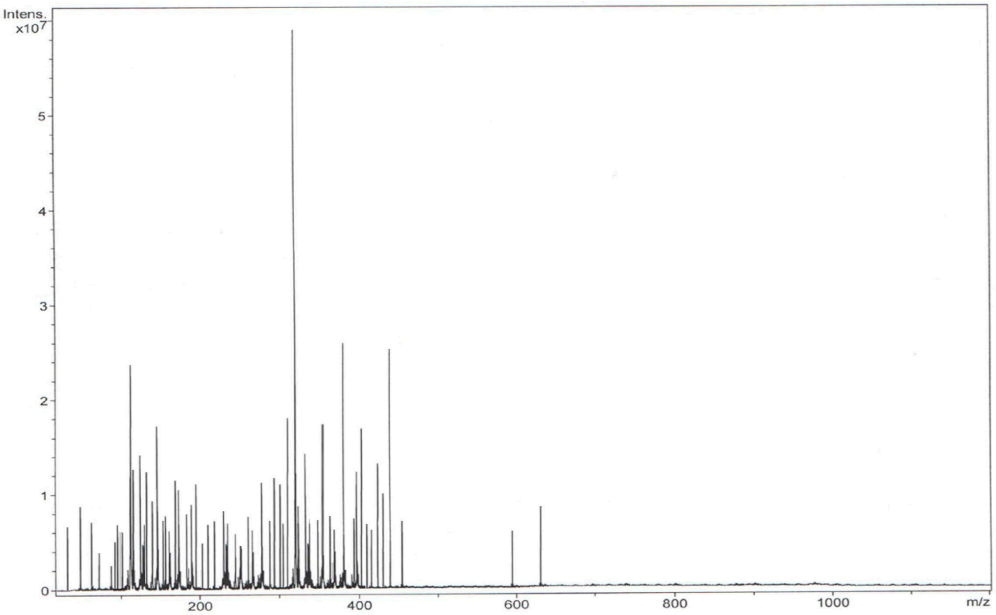

Figure S2. Mass spectrum of *E. cannabinum* sample.

Table S1. Results of TPC and selected antioxidant assays for samples before and after encapsulation.

Sample	TPC (mg GAE/g)	FRAP (mM Fe^{2+})	DPPH IC_{50} (mg/mL)
E. cannabinum	8.17±0.024	31.46±0.021	2.88±0.017
EC-AuNPs system	8.30±0.026	33.36±0.012	2.23±0.031
MEC carrier	9.15±0.019	35.93±0.018	2.51±0.110
MEC-AuNPs system	9.41±0.011	38.66±0.023	1.89±0.024

DPPH: 2,2-Diphenyl-1-picrylhydrazyl; FRAP: Ferric reducing antioxidant power; GAE: Gallic acid equivalents; IC_{50}: Half-maximal inhibitory concentration; TPC: Total phenolic content.
